# Diverged Early From CtpB and CtpC, CtpA Has Evolved to Process D1 Precursor in Oxygenic Photosynthetic Organisms

**DOI:** 10.3389/fpls.2021.676036

**Published:** 2021-04-30

**Authors:** Weidong Chang, Chenggang Li, Zheng Cui, Wei Li, Haifeng Song, Han Chang, Weihan Fu, Chunyu Wang, Ting Huang, Yixin Luo, Yelin Shan, Yuhua Wang, Fei Wang, Min Xu, Aigen Fu

**Affiliations:** ^1^Chinese Education Ministry’s Key Laboratory of Western Resources and Modern Biotechnology, Northwest University, Xi’an, China; ^2^Key Laboratory of Biotechnology Shaanxi Province, College of Life Sciences, Northwest University, Xi’an, China

**Keywords:** CtpA, CtpB, CtpC, C-terminal peptidase, D1 maturation, photosynthesis

## Abstract

C-terminal peptidase (Ctp) cleaves the C-terminal extension of the D1 precursor (pD1) to form mature D1. Among the three homologs CtpA, CtpB, and CtpC in photosynthetic organisms only the first is capable of processing pD1 while the roles of CtpB and CtpC remain elusive. Phylogenetic analysis of Ctps from photosynthetic organisms revealed that CtpA has diverged early from CtpB and CtpC during evolution implying distinct roles for the Ctps. Analysis of Arabidopsis Ctp-deficient mutants revealed that pD1 processing was not affected in *atctpb*, *atctpc*, or *atctpbatctpc* mutants, demonstrating that AtCtpA, not AtCtpB or AtCtpC, is responsible for cleaving the pD1 C-terminal extension. Ectopic expression of *CtpAs* from *Synechococcus elongatus*, *Chlamydomonas reinhardtii*, and *Physcomitrella patens* in *atctpa* rescued the lethal phenotype of the mutant indicating that SeCtpA, CrCtpA, and PpCtpA could process pD1 in Arabidopsis. Enzyme activity assays showed that PpCtpA and CrCtpA could convert pD1 into mature D1 *in vitro*. In contrast, expressing CtpB or CtpC from Arabidopsis, *C. reinhardtii*, or *P. patens* in *atctpa* did not rescue its D1 maturation deficiency, and enzyme activity assays also showed that neither CtpB nor CtpC could process pD1 *in vitro.* Taken together, we conclude that the function of pD1 processing by CtpA is conserved in photosynthetic organisms. It is possible that among other factors CtpA developed this function to initiate the formation of the oxygenic D1/D2 type PSII complex during evolution whereas CtpB or CtpC have other roles that are still unclear.

## Introduction

Photosystem II (PSII), a multimeric protein complex in the thylakoid membrane, catalyzes the light-driven oxidation of water and the reduction of plastoquinone (Ferreira et al., 2004; Wei et al., 2016). PSII assembly is a highly complex and coordinated process, starting with the formation of the PSII reaction center complex (including D1, D2, PsbE, F, and I), followed by the attachment of CP47 pre-complex and CP43 pre-complex with incorporating Low-molecular mass (LMM, including PsbH, K, L, T, Z, and 30) subunits to form monomeric PSII, and finally ending with the assembly of oxygen-evolving complex (OEC, including Mn_4_CaO_5_ cluster, PsbO, V, and U) and dimerization of the PSII complex (Komenda et al., 2012; Nickelsen and Rengstl, 2013). PSII is prone to photodamage under excessive light conditions with the D1 subunit as the primary damaged site. Once D1 is damaged, it must be removed and replaced with a newly synthesized subunit to rebuild a functional PSII (Adir et al., 2005; Järvi et al., 2015; Lu, 2016). Biogenesis and rapid turnover of D1 is crucial for PSII assembly and repair in oxygenic photosynthetic organisms.

D1, a thylakoid membrane protein, harbors five transmembrane α-helices, with its N-terminus in the stroma and the C-terminus in the thylakoid lumen (Sharma et al., 1997; Chi et al., 2012). Usually, there are three copies of *psbA* (the gene for D1) in prokaryotic cyanobacteria and a single copy in the chloroplast genome of photosynthetic eukaryotes (Golden et al., 1986; Nixon et al., 1991). In most oxygenic photosynthetic organisms, D1 is synthesized as a precursor protein (pD1) with a short C-terminal extension. After integration into the thylakoid membrane, the pD1 C-terminal extension is removed by a carboxyl-terminal peptidase (Ctp) to generate mature D1 (mD1) which provides a docking site for the manganese cluster of the oxygen evolving center (Trost et al., 1997; Roose and Pakrasi, 2004; Che et al., 2013). The pD1 C-terminal extension usually comprises 8 or 9 amino acids (aa) in eukaryotes while it is longer (usually 16 aa) in prokaryotes and requires two-step cleavage (Inagaki et al., 2001b). However, it is often absent in D1 from photosynthetic organisms that arouse through a secondary or tertiary endosymbiosis (Satoh and Yamamoto, 2007). Removing the nucleotide sequence of the extension did not affect the correct assembly of D1 into PSII (Nixon et al., 1992). The extension is not essential but improves a fitness of cyanobacterium *Synechocystis* sp. PCC 6803 lacking the extension (Ivleva and Shestakov, 2000). D1 is highly conserved in oxygenic photosynthetic organisms while the C-terminal extension of pD1 is quite divergent (Nixon et al., 1991; Satoh and Yamamoto, 2007). Ectopic expression of pD1 from the monocot *Poa annua* in a *Synechocystis* sp. PCC 6803 strain lacking *psbA* yielded a functional PSII, showing that pD1 from higher plants can be correctly processed and play a normal function in cyanobacteria (Nixon et al., 1991).

In general, oxygenic photosynthetic organisms possess three Ctp homologs, CtpA, CtpB, and CtpC (Satoh and Yamamoto, 2007). Disruption of CtpA blocked pD1 processing and resulted in a loss of PSII activity in *Synechocystis* sp. PCC 6803 (Anbudurai et al., 1994). A mutant in the green alga *Scenedesmus obliquus* with a deficiency in CtpA was unable to process pD1 and showed a non-photosynthetic low fluorescent phenotype (Trost et al., 1997). D1 remained in the pD1 form in an Arabidopsis mutant lacking AtCtpA and the mutant had a growth arrest phenotype indicating that AtCtpA is the enzyme responsible for processing the pD1 C-terminal extension in Arabidopsis (Che et al., 2013). Because the inactivation of CtpA usually resulted in a non-photosynthetic phenotype, CtpA was thought to be the enzyme responsible for cleavage of the pD1 C-terminal extension (Trost et al., 1997; Satoh and Yamamoto, 2007; Che et al., 2013). However, it was reported that a low level of pD1 processing could be catalyzed by other proteases in the absence of CtpA in *Synechocystis* sp. PCC 6803 (Komenda et al., 2007).

Structure analysis of CtpA from *S. obliquus* (SoCtpA) showed that it is a monomeric enzyme with three loosely packed folded domains (A, B, and C) (Liao et al., 2000). The B domain, a so called PDZ domain (a domain existing in PSD95, DlgA, and Zo-1 proteins in animal cells), was proposed to be the active site binding of the C-terminus of pD1, and the central S372/K397 catalytic dyad lies at the interface of the three domains (Liao et al., 2000). Site-directed mutagenesis of conserved residues of CtpA from *Synechocystis* sp. PCC 6803 demonstrated that 5 residues, S313, K338, D253, R255, and E316, are essential for its enzyme activity in which S313/K338 are equivalent to the S372/K397 catalytic dyad of SoCtpA (Inagaki et al., 2001a).

The optimal pH for CtpA is in the range of 5.5–6.0 with membrane-embedded pD1 as substrate, while pH 7.5–8.0 is preferred with a synthetic peptide as substrate. CtpA also shows a higher affinity to the membrane-embedded pD1 (*K*_*m*_ = ∼0.3 μM) than to the synthetic substrate (*K*_*m*_ = 0.3 mM) (Yamamoto et al., 2001). These differences suggest that CtpA probably interacts with some factors in thylakoid membranes which modulate the pH dependence of CtpA in response to physiological conditions (Yamamoto et al., 2001). It was reported that a TPR protein, named PratA, could facilitate D1 maturation through association with CtpA in cyanobacteria (Klinkert et al., 2004). A lumenal Psb27 homolog, LPA19, was also found to be involved in pD1 processing in Arabidopsis (Wei et al., 2010).

Compared to a handful of studies on CtpA, a very limited effort has been undertaken to dissect the roles of CtpB and CtpC. The Ctp gene expression and structure analysis suggested that they are play distinct roles in *Synechocystis* sp. PCC 6803 cellular processes and the structure differences exist in the putative membrane contact regions and in the active site environment (Jansèn et al., 2003). It was reported that a mutant strain of *Synechocystis* sp. PCC 6803 with inactivated CtpB grew photoautotrophically as wild type (Ivleva et al., 2002). In higher plants, disruption of AtCtpB did not result in any apparent defects in plant growth or alteration of thylakoid membrane proteins in Arabidopsis, although the *atctpb* mutant was more photoinhibited than wild type (Yin et al., 2008). Up to now, no investigation was conducted on CtpC in higher plants. It remains to be determined whether CtpB and CtpC function in processing of pD1.

In this study, we firstly analyzed the phylogenetic relationship between CtpA, CtpB, and CtpC from oxygenic photosynthetic organisms, and found that they diverged very early during evolution raising the possibility that they carry distinct functions. Then we examined their role in D1 maturation using genetic and biochemical approaches. We found that AtCtpB or AtCtpC, in contrast to AtCtpA, is unable to cleave the pD1 C-terminal extension in Arabidopsis. We further explored the pD1 processing activities of Ctps from three model organisms, including *Synechococcus elongatus* PCC 7942, *Chlamydomonas reinhardtii*, and *Physcomitrella patens*. We observed that all CtpAs could convert pD1 into D1 in Arabidopsis and *in vitro* while CtpB and CtpC could not. Therefore, we conclude that CtpA is the protease responsible for processing pD1 in oxygenic photosynthetic organisms whereas CtpB and CtpC never acquired the activity to process pD1 during evolution. We propose that the acquisition of the enzyme activity to process pD1 by CtpA may have been a critical event in initiating the formation of the oxygenic D1/D2 type PSII complex.

## Materials and Methods

### Primers

The detailed information of all the primers used in this study is listed in Supplementary Table 2.

### Plant Materials and Growth Conditions

Wild-type Arabidopsis Columbia-0 (Col-0) and five *ctp* mutants in Col-0 background were used in this study, including *atctpa* (SALK_056011, corresponding to the null mutant *atctpa-1* described in Che et al. (2013), *atctpb-1* (deletion mutant), *atctpb-2* (SAIL_169_G01), *atctpb-3* (SAIK_001461C), and *atctpc* (GK_359G01). All the mutant genotypes were confirmed by PCR and RT-PCR, and the *atctpa* mutant was further confirmed by immunoblotting. To test the functions of CtpA, CtpB, and CtpC from *Arabidopsis thaliana*, *P. patens*, *C. reinhardtii*, and *S. elongatus* PCC 7942, a series of transgenic lines were created in *atctpa* background. Ctp proteins expressed in these transgenic lines are either in native-form or in recombinant form by replacing their N-terminal chloroplastic transit peptide (TP) with the TP from AtCtpA (TP_AtCtpA_), a 126 aa peptide at the N-terminus (Schubert et al., 2002). The coding sequences (CDS) of these proteins fused with a C-terminal 2x HA tag were inserted downstream of the CaMV 35S promoter in the binary vector pRI101-AN (Takara, Japan) and transformed into +/*atctpa* heterozygous plants with the *Agrobacterium tumefaciens* strain GV3101 by the floral dip method (Clough and Bent, 1998). Transgenic plants were screened on 1/2 Murashige and Skoog (MS) medium supplemented with 1% sucrose and 25 mg/L kanamycin. PCR tests were performed to confirm the transgenic lines and host genotype at the *atctpa* locus.

Plants were grown at 23°C in a greenhouse under LL, NL or FL light conditions. For the analyses under LL light, plants were grown on 1/2 MS medium containing 1% sucrose with 25 μmol/s/m^2^ continuous light. For the analyses under NL light conditions, plants were grown in soil under 80 μmol/s/m^2^ continuous light for 3 weeks. For the analyses under FL light condition, plants were firstly grown in soil under NL for 1 week and then transferred under fluctuating light for 2 weeks. For the analyses under HL light conditions, plants were first grown in soil under NL for 2 weeks and then transferred to 350 μmol/s/m^2^ continuous light for 1 week.

### RNA Extraction, RT-PCR, and Real Time qRT-PCR

Total RNA was extracted from the leaves of 3-weeks-old plants with TRIzol^TM^ Reagent (Thermo Fisher Sci., United States), and treated with Turbo DNA-free kit (Invitrogen, United States) to remove genomic DNA contamination. Then the first-strand cDNA was synthesized using 1 μg of DNA-free RNA sample with the PrimeScript II first strand cDNA Synthesis Kit (Takara, Japan). All the experiments above were performed according to the manufacturer’s instructions.

For semi qRT-PCR, the first strand cDNA samples were diluted two times and 1 μl diluted sample was used as template for each PCR reaction. To test the expression levels of native genes, primers were designed based on their CDS except for the forward primer of AtCtpA gene located in the 5’UTR region before the T-DNA insertion site (see Supplementary Figure 2A); to test the expression levels of transgenic genes, at least one out of two primers was designed based on sequences from the vector pRI101-AN. *Actin 2* (*AT3G18780*) was used as control. For the qRT-PCR assay, the first strand cDNA samples were diluted 40 times and 1 μl diluted sample was used for each qPCR reaction. The qPCR assays were conducted on a LigthCycler 480 II instrument (Roche, Switzerland) using 2x SYBR Green qPCR Master Mix (Takara, Japan). Three replicates were carried out for each sample. The *GAPDH* (*AT3G26650*) gene was used as internal control for primary data normalization and gene expression levels relative to AtCtpA were calculated by the 2^–ΔΔCt^ method.

### Immunoblot Analysis and Antibodies

Denatured protein samples in 1x SDS loading buffer were separated on 12% SDS-PAGE gels or, for detecting D1, on 10% SDS-PAGE gels containing 8 M urea. The separated proteins were transferred to nitrocellulose membranes which were subsequently incubated with corresponding primary antibody overnight in 4°C. After that, the membranes were washed three times with 1x TBST and incubated with goat anti-rabbit IgG conjugated with horseradish peroxidase (HRP) (Bioworld, United States) with a dilution of 1:10,000. Finally, the chemiluminescent signals were generated using eECL Western Blot Kit (CWBIO, China) and captured with a CCD camera (Tanon 5200, China).

The HA primary antibody (#T506) was purchased from Signalway Antibody Company (United States). The polyclonal antibodies of ClpC and PC were obtained from Cocolico Biotechnical Company (United States). The polyclonal antibodies of α-AtCtpA, α-D1, and α-pD1 tail were generated by immunizing rabbits with GST fused mature AtCtpA (amino acids 127–515), pD1_304__–__353_ peptide, and pD1_345__–__353_ peptide, respectively. For examining D1 in plants, the α-D1 and α-pD1 tail crude antisera were used directly at a dilution of 1:2,000, but for Ctp enzyme activity assays, they were applied after affinity purification against peptide pD1_304__–__353_.

### Chloroplast Isolation and Sub-Fractionation and BN-PAGE

Chloroplast isolation was performed as previously described with slight modifications (Perry et al., 1991; Kauss et al., 2012). In brief, 3-weeks-old Arabidopsis rosette leaves were homogenized in buffer (450 mM D-sorbitol; 20 mM Tricine-KOH, pH 8.4; 10 mM EDTA; 10 mM NaHCO_3_; 0.1% BSA). Homogenized solution was filtered through two layers of Miracloth and the filtrate was centrifuged at 1,000*g* for 10 min at 4°C. Pellets were collected and resuspended with 2 ml resuspension buffer (300 mM D-sorbitol, 20 mM; Tricine-KOH, pH 8.4; 2.5 mM EDTA; 5 mM MgCl_2_). Suspensions were gently overlaid on a two-step percoll gradient composed of 5 ml 40% supertratum and 3 ml 80% substratum percoll, and then centrifuged at 4,000*g* for 20 min at 4°C. Intact chloroplasts were collected from the interface between the 40 and 80% percoll layers with cut tips and washed twice with resuspension buffer for further chloroplast subfractionation and BN-PAGE analyses.

Chloroplast subfractionation was performed as follow: Intact chloroplasts were incubated in 200 μl of lysis buffer (10 mM Hepes-KOH, pH 8.0) on ice for 10 min at a final concentration of 0.5 mg/ml chlorophyll, and then centrifuged at 20,000*g* for 10 min at 4°C. The supernatants containing stroma subfractions were pipetted to new tubes. The pellets were further resuspended in 200 μl of lysis buffer with 0.05% DM (*nN*-Dodecyl β-D-maltoside, Sigma-Aldrich, United States) and incubated on ice for 5 min. Then, the lysates were centrifuged at 20,000*g* for 1 h at 4°C to separate thylakoid lumen and thylakoid membranes, which were present in the supernatant and pellet fractions, respectively. The thylakoid membranes in pellets were resuspended in 200 μl of lysis buffer after washed twice to remove residual lumen proteins. The obtained chloroplast subfractions were used for further immunoblot analysis.

For BN PAGE analysis, intact chloroplasts were resuspended in 50BTH40G buffer (50 mM Bis Tris-HCl pH 7.0, 40% Glycerol) to a final chlorophyll concentration of 1 mg/ml. Resuspended chloroplasts were treated with 1% DM on ice for 5 min, and centrifuged at 10,000*g* for 10 min at 4°C. Then, supernatants were mixed in 1x BN loading buffer (0.5% Serva G; 7.5% Sucrose; 10 mM Bis Tris-HCl; 50 mM 6-amino-Caproic acid; pH 7.0), and loaded on a BN gradient gel (5–13%) which was prepared as described (Järvi et al., 2011). Electrophoresis was conducted at 110 V for 5 h at 4°C with Mini-PROTEAN^®^ Tetra System (BIO-RAD, United States) with 50 mM Bis Tris-HCl (pH 7.0) as the anode buffer and 50 mM Tricine/15 mM Bis Tris-HCl as the cathode buffer. During electrophoresis, the initial cathode buffer containing 0.01% Serva Blue G dye (Sigma-Aldrich, United States) was replaced with the same buffer lacking the dye halfway through the run.

### *In vitro* Ctp Enzyme Activity Assay

The CDS of proteins used for the enzyme activity assays were cloned downstream of the GST tag in the expression vector pGEX4T-3 (GE Healthcare, United States), including the mature peptides of Ctps and the C-termini of pD1s. The vectors were transformed into *Escherichia coli* BL-21 (DE3) and induced to express target proteins at 16°C for 20 h with 0.1 mM Isopropyl-beta-D-thiogalactoside (IPTG). Afterward bacterial cells were collected by centrifugation (3 min, 7,000*g*, 4°C) and washed twice with PBS (140 mM NaCl, 2.7 mM KCl, 10 mM Na_2_HPO_4_, 1.8 mM KH_2_PO_4_). Then the cells were resuspended in lysis buffer (0.1 mg/ml lysozyme, 1 mM PMSF, 0.1% Triton x-100, dissolved in PBS), incubated at 4°C for 30 min, and broken by sonication. After centrifugation, the GST fusion proteins were purified from supernatants through affinity chromatograph with Glutathione sepharose^TM^ 4B (GE Healthcare, United States) according to the manufacturer’s instruction.

*In vitro* Ctp enzyme activity assays were conducted in 200 μl reaction systems in 100 mM MES (pH 5.0), 100 mM HEPES (pH 7.0), or 100 mM CHES (pH 9.0) (Yamamoto et al., 2001; Che et al., 2013). Ten micrograms of GST-Ctps were used for the proteolytic reaction with recombinant or native pD1 substrates, that is, GST-AtpD1/SepD1s (10 μg) or 0.1% DM-treated thylakoid membranes (50 μg of chlorophyll) from the *atctpa* mutant. Proteolytic reactions were carried out at 25°C and stopped at time points of 0, 5, 10, 20, and 60 min by mixing with an equal volume of 2x SDS loading buffer. The treated protein samples were further subjected to immunoblot analysis and examined against purified α-D1 and α-pD1 tail antibodies.

### Sequence Retrieval, Alignment and Phylogenetic Tree

BlastP searches from NCBI^1^ or JGI^2^ at the threshold of ≥70% coverage and ≤1e-30 e-value) retrieved 286 Ctp proteins from 93 photosynthetic organisms in Cyanophyta (20), Chlorophyta (18), Bryophyta (3), Tracheophyte (1), Angiosperm (49), Chlorarachniophyta (1), and Dinophyta (1) (Supplementary Table 1). Two tail-specific protease (Tsp) proteins from *E. coli* were used as an outgroup. To construct phylogenetic trees, all protein sequences were initially compared in multiple sequence alignments using ClustalW program of MEGA6. As noted, the AtCtps protein used in this analysis were the mature forms. Subsequently, the output of alignment was used to build a phylogenetic tree by MEGA6 using the Neighbor-Joining method with the bootstrapping value set at 1,000 replications and the Maximum-Likelihood method with the bootstrapping value set at 500 replications (Felsenstein, 1985; Saitou and Nei, 1987; Tamura et al., 2013).

### Three-Dimension Structure Analyses

The pdb file of the SoCtpA three-dimensional structure was obtained from the PDB database^3^ s. Three-dimension structures of AtCtpA, AtCtpB, and AtCtpC were predicted by I-TASSER (Zhang, 2009; Roy et al., 2012; Yang and Zhang, 2015; Zhang et al., 2017). These four pdb files were uploaded in the Visual Molecular Dynamics program (Humphrey et al., 1996) to generate the presented structures.

### Accession Numbers

All Ctp protein accession numbers used in this study are listed in Supplementary Table 1.

## Results

### CtpA, CtpB, and CtpC Diverged Early During the Evolution of Oxygenic Photosynthetic Organisms

It is interesting that most oxygenic photosynthetic organisms encode three Ctps but only CtpA is critical for cleaving the pD1 C-terminal extension (Ivleva et al., 2002; Satoh and Yamamoto, 2007; Che et al., 2013). We postulated that CtpA, CtpB, and CtpC have developed distinct functions during evolution. To explore this hypothesis, we conducted a phylogenetic analysis of Ctps from oxygenic photosynthetic organisms, including 20 Cyanophyta species, 18 Chlorophyta species, 3 Bryophyta species, 1 Tracheophyte species, 49 Angiosperm species, 1 Chlorarachniophyta species, and 1 Dinophyta species (Supplementary Table 1). Two tail specific proteases (Tsp) from *E. coli* served as the outgroup control (Silber et al., 1992).

**FIGURE 1:**
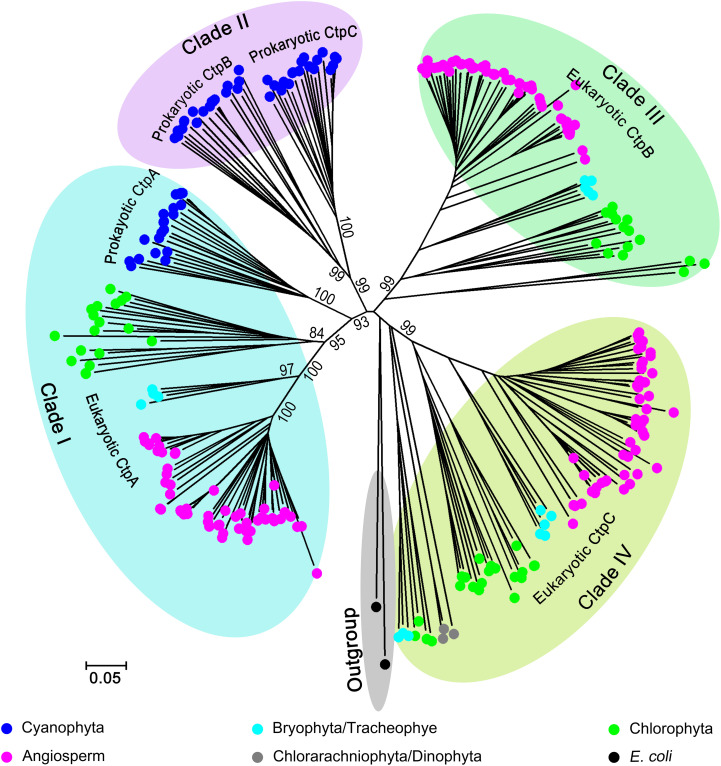
Phylogenetic analysis of *C-terminal peptidase* (CTP) family. Two hundred and eighty-six Ctp proteins were retrieved from 93 oxygenic photosynthetic species, including Cyanophyta (20), Chlorophyta (18), Bryophyta (3), Tracheophyte (1), Angiosperms (49), Chlorarachniophyta (1), and Dinophyta (1). For the phylogenetic analysis, the polypeptide sequences were subjected to the multiple sequence alignment (MSA) analysis with ClustalW. The phylogenetic tree was constructed with MEGA6 base on the MSA profile using the Neighbor-Joining method (Saitou and Nei, 1987) and a 1,000 bootstrap resampling value (Felsenstein, 1985). Two tsp proteins from *Escherichia coli* were used as the outgroup.

The phylogenetic analysis indicated that Ctp proteins evolved from a single ancestor and followed a complex evolutionary pathway to generate the paralogs in photosynthetic organisms (Figure 1 and Supplementary Figure 1). Overall, the Ctp family can be clustered into four major clades (Figure 1 and Supplementary Figure 1). In detail, clade I includes eukaryotic and prokaryotic CtpAs; clade II encompasses prokaryotic CtpBs and CtpCs; clade III harbors eukaryotic CtpBs and clade IV consists of eukaryotic CtpCs.

All CtpAs from photosynthetic organisms are clustered in a monophyletic clade (Clade I) suggesting that the ancestor of CtpA emerged early in evolution, and prokaryotic and eukaryotic CtpAs should share a conserved function. Unexpectedly, the phylogenetic analysis showed that CtpBs and CtpCs from prokaryotes and eukaryotes have undergone different and complex evolutionary changes. Clade II of prokaryotic CtpB and CtpC shows a close relationship with Clade I of CtpA indicating that CtpA and prokaryotic CtpB or C share a common ancestor and have diverged early during evolution of prokaryotic photosynthetic organisms (Figure 1 and Supplementary Figure 1). On the other hand, clade III of eukaryotic CtpB and clade IV of eukaryotic CtpC evolved in independent lineages distinct from Clade I and II suggesting that eukaryotic CtpB or C are not derived from the most recent common ancestor shared by prokaryotic CtpB or C and CtpA (Figure 1 and Supplementary Figure 1). The phylogenetic analysis showed that CtpA, CtpB, and CtpC have diverged at an early stage of evolution, raising the possibility that CtpA, CtpB, and CtpC may carry distinct functions.

### Loss of CtpB and CtpC or Both Does Not Alter Plant Growth and pD1 Processing in Arabidopsis

**FIGURE 2:**
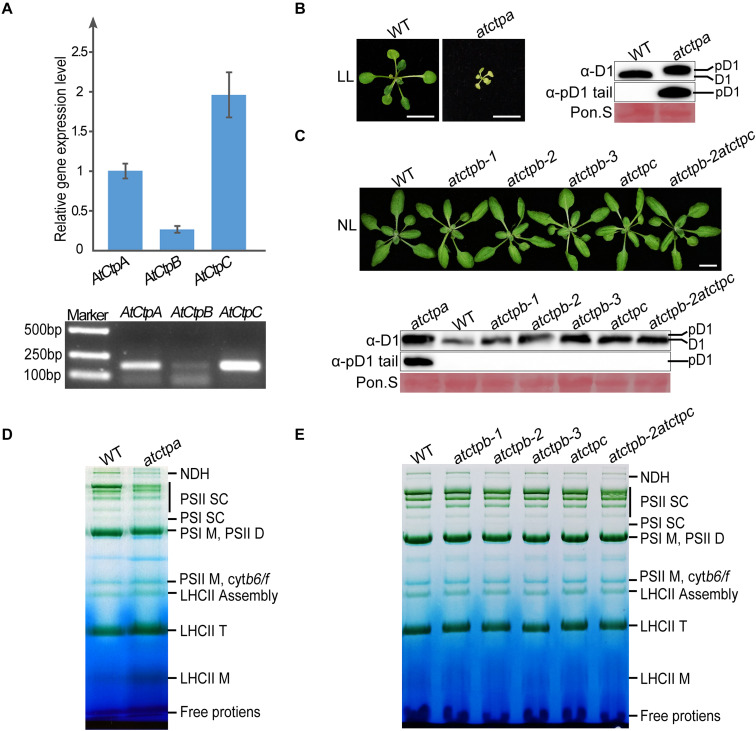
Characterizations of *atctpa*, *atctpb*, and *atctpc* mutants. **(A)** AtCtp transcript levels in 3-weeks-old wild-type (WT) rosette leaves. Upper panel shows quantitative RT-PCR results; lower panel shows semi quantitative qRT-PCR analysis. For qRT-PCR, *GAPDH* (*AT3G26650*) was used as internal control for primary data normalization. Error bars indicate standard deviations (SD) from three biological replicates. **(B)** Characterization of *atctpa* mutant. WT and *atctpa* were grown on 1/2 MS with 1% sucrose under continuous low light (LL, 25 μmol/s/m^2^) for 3 weeks (Left). Scale bars represent 1 cm. The D1 processing status was analyzed by immunoblotting with α-pD1 tail (for pD1) and α-D1 (for D1 and pD1) antibodies (Right). The filters stained with Ponceau S (Pon. S) were used as loading controls. **(C)** Characterizations of *atctpb* and *atctpc* mutants. WT, single mutants (*atctpb*-1, -2, -3, and *atctpc*), and double mutant (*atctpb*-2*atctpc*) were grown in soil under continuous normal light (NL, 80 μmol/s/m^2^) for 3 weeks (Upper). Scale bars denote 1 cm. The D1 processing statuses were analyzed by immunoblot against α-pD1 tail (for pD1) and α-D1 (for D1 and pD1) antibodies (Lower). The pD1 protein from *atctpa* mutant in panel (B) were used as positive control. The filters stained with Ponceau S (Pon. S) were used as loading controls. (D,E) BN-PAGE analyses of *atctpa*, *atctpb*, and *atctpc* mutants. The plants in panel **(D)** were grown on 1/2 MS under continuous LL light for 3 weeks. The plants in panel **(E)** were grown in soil under continuous NL light for 3 weeks. Thylakoid membrane protein complexes (12 μg chlorophyll) were loaded on BN-PAGE gels for electrophoresis.

To examine the roles of Ctps, we first analyzed the functions of Ctps in Arabidopsis. There are three Ctp members in Arabidopsis, including AtCtpA (*At4g17740*), AtCtpB (*At3g57680*), and AtCtpC (*At5g46390*). RT-PCR analysis revealed that all *Ctp* genes are expressed in the leaf tissue implying potential physiological roles of these genes (Figure 2A).

We obtained three *atctpb* (*atctpb-1*, *-2*, *and -3*) and one *atctpc* null mutant, of which *atctpb-1* was created by the CRISPR-Cas9 method while the others are T-DNA insertion mutants (Supplementary Figure 2). In contrast to the severe growth deficiency of the *atctpa* mutant (Che et al., 2013; Figure 2B), all *atctpb* and *atctpc* single mutants and the *atctpb–2atctpc* double mutant grew as wild-type plants (WT) under normal light (NL) growth condition (Figure 2C). Similarly, no obvious difference was observed between *atctpb*, *atctpc*, and *atctpb-2atctpc* and WT under fluctuating light (FL) and high light (HL) conditions (Supplementary Figure 3).

Do CtpB and CtpC have a role in pD1 processing? To monitor pD1 processing in planta, we produced two antibodies. The α-D1 antibody, raised against the C-terminal 50 aa fragment of pD1, can detect both mD1 and pD1; the other α-pD1 tail antibody against the 9 aa C-terminal extension of pD1 can only detect pD1. D1 protein of WT Arabidopsis was detected by α-D1 but not by α-pD1 tail, while D1 in *atctpa*, which is present in the pD1 form, was identified by both α-D1 and α-pD1 tail (Figure 2B). The band representing pD1 in *atctpa* is slightly larger than that of mD1 in WT because of the presence of the C-terminal tail. These results demonstrate that both antibodies work efficiently and specifically. We therefore used them to investigate the D1 processing status in the *atctpb*, *atctpc*, and *atctpb-2atctpc* mutants under NL, FL, and HL conditions. The immunoblot results showed that only mD1 but no pD1 could be observed in these mutants, suggesting that pD1 processing is not affected by the deficiency of AtCtpB, AtCtpC, or of both (Figure 2C; Supplementary Figure 3).

Additionally, we analyzed the composition of photosynthetic complexes of these mutants by blue native PAGE (BN-PAGE). The pD1 C-terminal processing is critical for the assembly and repair of photo-damaged PSII, and blocking pD1 processing results in a large change of the PSII supercomplex (PSII SC) profile (Che et al., 2013). The *atctpa* mutant accumulated much lower amounts of PSII SC compared to WT (Figure 2D). In contrast, single and double mutants of *atctpb* and *atctpc* exhibited BN-PAGE patterns very similar to those of WT (Figure 2E) indicating that the assembly of PSII SC is not disturbed in these mutants. These results show that the deficiency of AtCtpB and/or AtCtpC does not affect pD1 processing in Arabidopsis, and they confirm that AtCtpA is the only enzyme responsible for this processing event (Che et al., 2013).

### CtpAs From Other Model Photosynthetic Organisms Can Process pD1 in Arabidopsis

**FIGURE 3:**
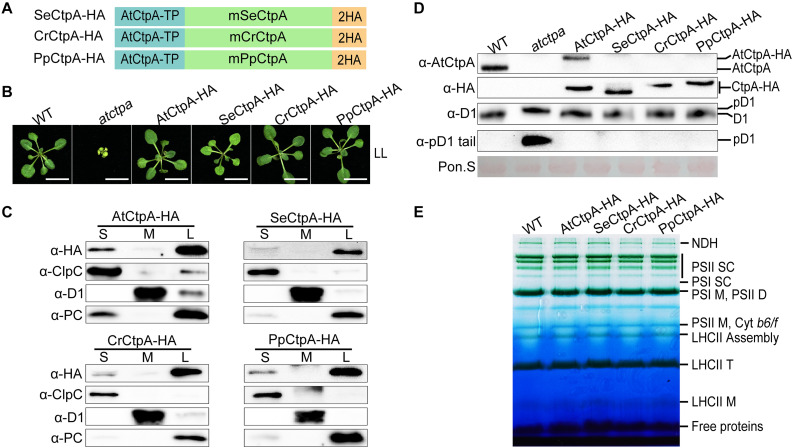
Ectopic expression of CtpA from lower photosynthetic organisms in the *atctpa* mutant. **(A)** Schematic diagrams of fusion proteins *SeCtpA-HA* (from *Synechococcus elongatus*), *CrCtpA-HA* (from *Chlamydomonas reinhardtii*), and *PpCtpA-HA* (from *Physcomitrella patens*). *AtCtpA-TP* is the chloroplast *TP* of AtCtpA protein; mPpCtpA, mCrCtpA, and mSeCtpA are the mature proteins; 2HA indicates a double hemagglutinin tag. **(B)** Phenotype of *atctpa* plants transformed with CaMV 35S promoter-driven *SeCtpA-HA*, *CrCtpA-HA*, and *PpCtpA-HA*. Plants were grown on 1/2 MS medium with 1% sucrose under continuous LL light (25 μmol/s/m^2^) for 3 weeks. At least five transgenic lines per construct were examined. Scale bars represent 1 cm. **(C)** Chloroplast sub-compartment distribution of AtCtpA, SeCtpA, CrCtpA, and PpCtpA. Intact chloroplasts from transgenic plants expressing *AtCtpA-HA*, *SeCtpA-HA*, *CrCtpA-HA*, and *PpCtpA-HA* were fractionated into stroma (S), thylakoid membrane (M), and thylakoid lumen (L). The distribution of CtpA in the three fractions was examined by immunoblotting with a-HA and compared with the thylakoid lumen protein PC, the thylakoid membrane protein D1, and the stromal protein ClpC. **(D)** D1 processing status in *atctpa* plants transformed with CaMV 35S promoter-driven *SeCtpA-HA*, *CrCtpA-HA*, and *PpCtpA-HA*. D1 processing in plants in panel (B) was analyzed by immunoblotting using the following antibodies: α-AtCtpA for AtCtpA, α-HA for ectopically expressed *CtpA-HA*, α-D1 for D1 and pD1, and α-pD1 tail for pD1. The filters stained with Ponceau S (Pon. S) were used as loading control. **(E)** BN-PAGE analyses of *atctpa* plants transformed with CaMV 35S promoter-driven *SeCtpA-HA*, *CrCtpA-HA*, and *PpCtpA-HA*. The transgenic and WT plants were grown in soil under continuous NL light (80 μmol/s/m^2^) for 3 weeks before analyses. Thylakoid membrane protein complexes (12 μg chlorophyll) were loaded on a BN-PAGE gel for electrophoresis.

The analysis of the AtCtp deficient mutants revealed that AtCtpA is the only enzyme for pD1 processing in Arabidopsis. Considering that CtpA of prokaryotes and eukaryotes share a recent common ancestor (Figure 1 and Supplementary Figure 1), we postulated that CtpA may be the only enzyme that acquired the capacity of processing pD1 during evolution and that this function should be conserved in oxygenic photosynthetic organisms. To test the functional conservation of CtpA, we ectopically expressed CtpA from three model photosynthetic organisms, including the cyanobacterium *S. elongatus* PCC 7942, the green alga *C. reinhardtii*, and the moss *P. patens*, in Arabidopsis to examine whether they could process pD1 in Arabidopsis. More specifically, the mature CtpA proteins with a C-terminal 2x HA tail was fused to the C-terminus of the AtCtpA TP and expressed in the *atctpa* mutant (Figure 3A). Because the *atctpa* mutant is lethal, we first introduced the designed genes into AtCtpA*/atctpa* heterozygous plants, then segregated the offspring and obtained transgenic plants with the *atctpa* background.

Under low light (LL) growth conditions, ectopic expression of *SeCtpA-HA* (from *S. elongatus*), *CrCtpA-HA* (from *C. reinhardtii*), and *PpCtpA-HA* (from *P. patens*) was able to rescue the lethal phenotype of the *atctpa* mutant, although transgenic *atctpa* plants expressing *SeCtpA-HA* were light green and slightly smaller compared with WT (Figure 3B). Protein sub-chloroplastic localization studies revealed that all the exogenous CtpAs were present in the thylakoid lumen as PC (plastocyanin) (Figure 3C) demonstrating that the TP of AtCtpA can direct the fused CtpA proteins into the thylakoid lumen. Immunoblot analysis showed that the D1 protein is present in the mD1 form in these transgenic plants, as in WT and *AtCtpA-HA* complemented *atctpa* plants (Figure 3D). Thylakoid membrane photosynthetic complexes were also restored to WT levels by expressing these three foreign CtpAs (Figure 3E). Notably, the AtCtpA antibody could not detect *SeCtpA-HA*, *CrCtpA-HA*, or *PpCtpA-HA* (Figure 3D), probably because of the low sequence homology between AtCtpA and its foreign counterparts. Overall, these results show that CtpAs from three evolutionary diverse photosynthetic organisms are able to correctly process Arabidopsis pD1, and confirm that the function of CtpA is well conserved from photosynthetic bacteria to higher plants, although they have a low sequence identity (indicate 47.44%).

### AtCtpA Exhibits the Highest Efficiency to Process Arabidopsis pD1 and SepD1

**FIGURE 4:**
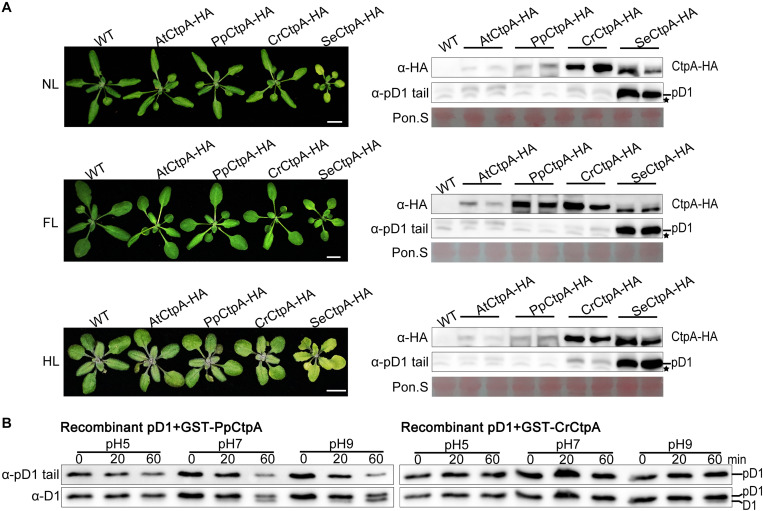
Protease activity of CtpA for AtpD1 processing from four oxygenic photosynthetic organisms. **(A)** Characterizations of the phenotypes (left) and pD1 processing status (right) in transgenic *atctpa* plants expressing *AtCtpA-HA*, *PpCtpA-HA*, *CrCtpA-HA*, and *SeCtpA-HA* under different light conditions. Plants were gown in soil under continuous light as follows: NL, normal light (80 μmol/s/m^2^) for 3 weeks; FL, normal light for 1 week, then fluctuating light for 2 weeks (the light intensity changes are shown in Supplementary Figure 3A); HL, normal light for 2 weeks and then high light (350 μmol/s/m^2^) for 1 week. Scale bars denote 1 cm. The pD1 processing in these transgenic plants was assessed by immunoblotting with α-HA (for ectopic expressed *CtpA-HAs*) and α-pD1 tail (for native pD1). Two plants were examined for each transgenic line. The filters stained with Ponceau S (Pon. S) were used as loading controls. Asterisks indicate non-specific bands. **(B)** *In vitro* protease activities of the *PpCtpA* and *CrCtpA* were tested with recombinant *AtpD1* substrate. Recombinant *AtpD1* was a fusion of GST with the C-terminal 50 aa fragment of *Arabidopsis thaliana* pD1. Proteolytic reactions were performed in pH 5.0, 7.0, and 9.0, respectively, stopped at the time points of 0, 5, 10, 20, and 60 min, and examined for the pD1 processing statuses by immunoblot against α-pD1 tail (for pD1) and α-D1 (for D1 and pD1) antibodies.

Eukaryotic PpCtpA and CrCtpA could fully substitute for AtCtpA function under low light growth condition while prokaryotic SeCtpA was significantly less efficient in cleaving the tail of Arabidopsis pD1. We further planted all the *CtpA-HA* transgenic plants under NL, FL, and HL light conditions and analyzed their phenotypes and the status of pD1 processing (Figure 4A).

Transgenic plants with *SeCtpA-HA* in *atctpa* background had a yellow dwarf but viable phenotype, especially under HL condition (Figure 4A, left), and immunoblot analysis indicated a large accumulation of pD1 in these plants under all three light conditions (Figure 4A, right). On the contrary, *atctpa* plants transformed with *CrCtpA-HA* and *PpCtpA-HA* grew as WT under all light conditions (Figure 4A, left). However, *atctpa* plants harboring *CrCtpA-HA* accumulated a large amount of pD1 when grown under HL (Figure 4A, lower right). D1 is prone to photodamage, and damaged D1 is rapidly replaced with newly synthesized D1 to assemble a repaired PSII complex (Baena-González and Aro, 2002; Adir et al., 2005; Edelman and Mattoo, 2008). The D1 maturation performed by CtpA is the key step in the PSII repair as it allows a quick restoration of oxygen evolution in the PSII with the new D1. The slight accumulation of pD1 in *atctpa* harboring *CrCtpA-HA* under HL indicates that CrCtpA processed Arabidopsis pD1 less efficiently than AtCtpA and PpCtpA, but more efficiently than SeCtpA.

**FIGURE 5:**
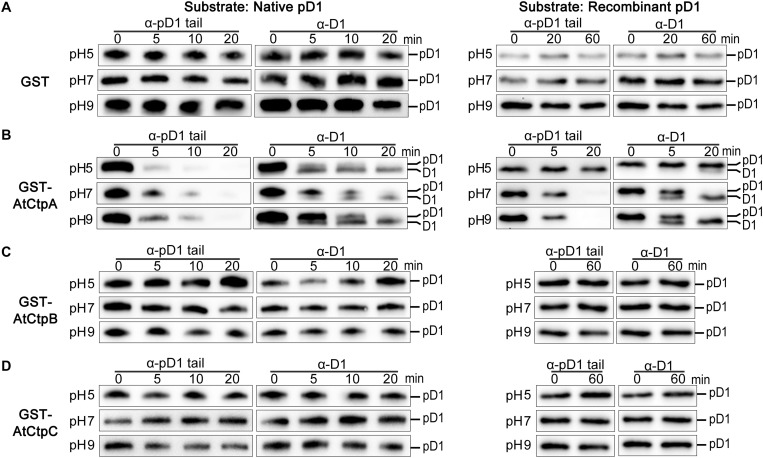
*In vitro* analyses of the AtCtpA **(B)**, AtCtpB **(C)**, and AtCtpC **(D)** protease activities. *In vitro* protease activity of AtCtpA **(B)**, AtCtpB **(C)**, and AtCtpC **(D)** was assayed with the substrates native pD1 (Left) and recombinant pD1 (Right). Free GST **(A)** was used as negative control. Native pD1 was from 1% DM detergent-treated thylakoid membranes extracted from the *atctpa* mutant; recombinant pD1 was recombinant *AtpD1*. Proteolytic reactions were performed as described in the legend of Figure 4.

We further examined the enzyme activities of CrCtpA and PpCtpA with recombinant Arabidopsis pD1 as substrate. Because we were not able to induce the expression of SeCtpA in *E. coli* cells, the activity test of SeCtpA was not performed. We used GST-CrCtpA and GST-PpCtpA in the enzyme assay (Supplementary Figure 7 and Figure 4B) that showed that the Ctp activity of PpCtpA is higher than CrCtpA. However, both PpCtpA and CrCtpA showed a much lower Ctp activity than AtCtpA. At pH 9.0, PpCtpA converted half of pD1 to mD1 in 60 min, while CrCtpA only converted trace amounts of pD1 in mD1 (Figure 4B). In comparison AtCtpA could actively convert all pD1 to mD1 in 20 min at pH 9.0 (Figure 5B, right). Therefore, the order of CtpA protease activity with the Arabidopsis pD1 substrate is: AtCtpA > PpCtpA > CrCtpA > SeCtpA.

**FIGURE 6:**
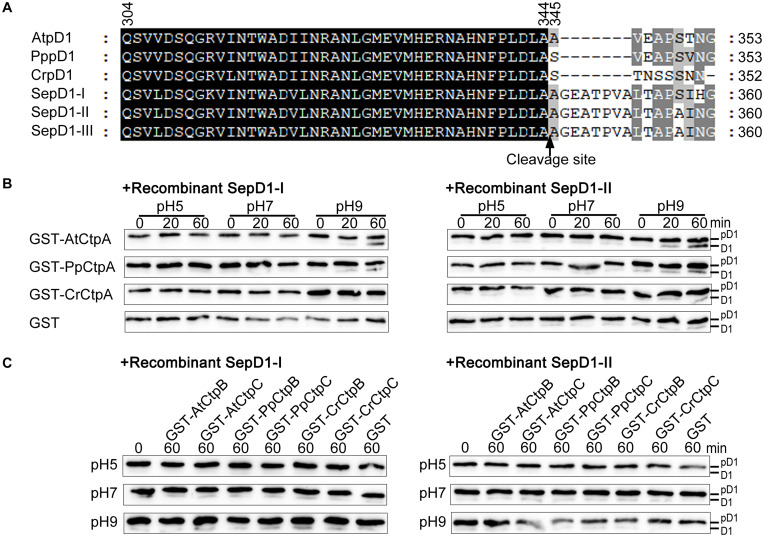
Protease activity of eukaryotic Ctp on prokaryotic pD1. **(A)** C-terminal sequence comparison of pD1 from four evolutionary lineages of oxygenic photosynthetic organisms. *AtpD1*, *A. thaliana* pD1; *PppD1*, *P. patens* pD1; *CrepD1*, *C. reinhardtii* pD1; *SepD1*-I, -II, and -III, *S. elongatus* PCC 7942 pD1-I, -II, and -III. The numbers indicate the positions of the corresponding amino acids in pD1. The C-terminal cleavage site between amino acids 344 and 345 is indicated by a vertical arrow. **(B)** *In vitro* protease activity assays of eukaryotic CtpA using as substrate recombinant *SepD1*-I (left) and *SepD1*-II (right), which were produced by fusing GST with the C-terminal 57 aa fragment of *SepD1*-I or *SepD1*-II. The experiments were conducted as described in the legend of Figure 4. **(C)** *In vitro* protease activity assays of eukaryotic CtpB and CtpC using as substrate recombinant *SepD1*-I (left) and *SepD1*-II (right).

As expected, AtCtpA showed the highest activity against the Arabidopsis pD1 substrate because of the co-evolutionary relationship between AtCtpA and its native substrate. If the co-evolutionary relationship is critical for CtpA activity, we wondered whether CtpA from higher organisms could function against a substrate from lower organisms. To answer this question, we switched the substrate from the C-terminus of Arabidopsis pD1 to that of *S. elongatus* pD1 in the *in vitro* enzyme assay. There are three *psbA* genes in the genome of *S. elongatus*, encoding SepD1-I, -II, and -III with a 16 aa C-terminal extension (Figure 6A). As the sequences of SepD1-II and -III are identical, we generated two recombinant cyanobacteria pD1 substrates, GST-SepD1-I and -II, in which the pD1 segment consists of the last 57 amino acids corresponding to the Arabidopsis pD1 region used in this study (Figure 6A).

Compared to Arabidopsis pD1, the processing activity of three eukaryotic CtpAs to both cyanobacterial pD1 substrates was inefficient; however, we could still observe the order of proteolytic activity as follows: AtCtpA > PpCtpA > CrCtpA (Figure 6B). AtCtpA digested less than half of pD1 to mD1 in 60 min at pH 9.0, while the mD1 signal produced by PpCtpA was weaker and the one by CrCtpA was the weakest (Figure 6B). It seems that CtpA protease activity increases along with the evolutionary lineage regardless of the source of the substrate.

### The Phenotype Analysis of *atctpa* Plants Carrying AtCtpB or AtCtpC Driven by 35S Promoter

In Arabidopsis, the absence of pD1 accumulation and the normal growth phenotype observed in *atctpb* and *atctpc* single and double mutants demonstrates that AtCtpB and/or AtCtpC are not required for pD1 processing *in vivo*. However, we cannot exclude the possibility that AtCtpB and AtCtpC could be active but expressed at a relatively lower level, which might be not enough to enable significant pD1 processing in chloroplasts.

**FIGURE 7:**
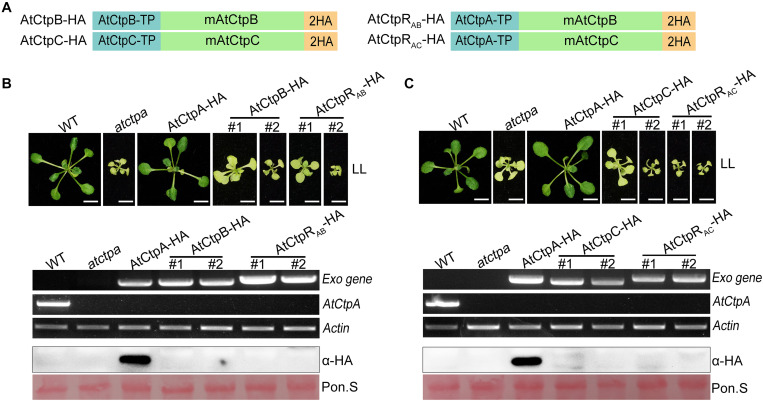
The phenotype of atctpa plants carrying AtCtpB or AtCtpC driven by 35S promoter. **(A)** Schematic diagrams of *AtCtpB-HA* or *AtCtpR*_*AB*_-*HA* and *AtCtpC-HA* or *AtCtpR*_*AC*_-*HA*. *AtCtpA-TP*, *AtCtpB-TP*, and *AtCtpC-TP* are the chloroplast transit peptides of the corresponding AtCtp proteins; mAtCtpB and mAtCtpC represent the mature proteins; 2HA indicates a double hemagglutinin tag. **(B,C)** Ectopic expressions of *AtCtpB-HA* or *AtCtpR_*AB*_-HA* and *AtCtpC-HA* or *AtCtpR_*AC*_-HA* in the *atctpa* mutant. CaMV 35S promoter-driven *AtCtpB-HA* or *AtCtpR_*AB*_-HA* and *AtCtpC-HA* or *AtCtpR_*AC*_-HA* were transformed into the *atctpa* mutant through the Agrobacterium-mediated method. Upper panels in **(B,C)** the plants were grown on 1/2 MS with 1% sucrose under continuous low light (LL, 25 μmol/s/m^2^) for 3 weeks; #1 and #2 indicate two independent transgenic lines. Scale bars represent 0.5 cm. Lower panels in **(B,C)** expression of exogenous transgenes (*Exo*) in transgenic plants were examined at RNA (top three images) and protein (bottom two images) levels by semi qRT-PCR and immunoblot analysis. For semi qRT-PCR analysis, expressions of the endogenous AtCtpA gene in transgenic plants was used for confirming backgrounds, and expression of the housekeeping gene *Actin 2* (*AT3G18780*) was used as quantity control. For immunoblot analyses, the filters stained with Ponceau S (Pon. S) were used as loading controls.

To test this assumption, we expressed AtCtpA, AtCtpB, and AtCtpC fused with a 2x HA tag in *atctpa*, in which these genes were driven by 35S promoter (Supplementary Figure 4A and Figure 7A). We found that *atctpa* plants carrying *AtCtpA-HA* grew normally like WT regardless of the expression level of *AtCtpA*-*HA* (Supplementary Figure 4). However, all *atctpa* transgenic plants expressing *AtCtpB-HA* or *AtCtpC-HA* had the same yellowish and growth arrest phenotype as *atctpa* (>100 T1 transgenic plants for each construct) (Figures 7B,C). RT-PCR assays showed that *AtCtpB-HA* and *AtCtpC-HA* were abundantly transcribed in these transgenic plants. However, immunoblot analysis revealed that *AtCtpB-HA* and *AtCtpC-HA* proteins did not accumulate (Figures 7B,C). We also examined the expression of *AtCtpB-HA* and *AtCtpC-HA* in transgenic plants in heterozygous or wild-type genetic background, and a similar pattern with abundant mRNA and undetectable protein was observed in these transgenic plants (Supplementary Figure 5).

In addition, to ensure correct localization of AtCtpB and AtCtpC in the thylakoid lumen, we created two new recombinant genes, *AtCtpR_*AB*_-HA* and *AtCtpR_*AC*_-HA* by replacing their native TP s with that of AtCtpA (Figure 7A). Even with these changes, *AtCtpR_*AB*_-HA* and *AtCtpR_*AC*_-HA* transgenes did not rescue the lethal phenotype of *atctpa.* Expression analysis revealed that the *AtCtpR_*AB*_-HA* and *AtCtpR_*AC*_-HA* genes are transcribed well in transgenic plants but the corresponding protein levels were below detection (Figures 7B,C). Analysis of *AtCtpR_*AB*_-HA* and *AtCtpR_*AC*_-HA* transgenic plants in heterozygous or wild-type genetic background showed a similar pattern of high mRNA level and undetectable protein in these transgenic plants (Supplementary Figure 5). We speculated that AtCtpB or AtCtpC maybe have a detrimental effect on Arabidopsis which limits their accumulation *in vivo.*

### AtCtpB and AtCtpC Cannot Cleave the C-Terminal of pD1 Substrate *in vitro*

AtCtpB and AtCtpC have a low sequence identity with AtCtpA, but they contain all the conserved amino acid residues essential for Ctp protease activity (Supplementary Figure 6A; Inagaki et al., 2001a). The structures of the three AtCtps predicted by I-TASSER program were very similar to that of SoCtpA, and the position and orientation of the essential catalytic dyad were almost identical in these three AtCtps (Supplementary Figure 6A) which still led us to suspect that AtCtpB and AtCtpC might possess the enzyme activity to process pD1. To test this possibility, we expressed and purified GST-AtCtpB and GST-AtCtpC fusion proteins in *E. coli* (Supplementary Figure 7), and performed the Ctp activity assay with two substrates: one was the thylakoid embedded pD1 prepared from *atctpa* plants, and the other was a recombinant pD1 produced by fusing GST with the C-terminal 50 aa fragment of pD1.

Free GST did not cause any change to native pD1 or recombinant pD1 substrate at different pH conditions as expected (Figure 5A). When GST-AtCtpA was used in the enzyme assay, the pD1 signal detected by the α-pD1 tail antibody gradually disappeared, and the mD1 signal detected by the α-D1 antibody progressively increased (Figure 5B) indicating that GST-AtCtpA could efficiently convert pD1 into mD1. The proteolytic activity of GST-AtCtpA showed a different pH dependency with different substrates. The optimal pH for native pD1 and synthetic recombinant pD1 is 5.0 and 9.0, respectively (Figure 5B).

In contrast to GST-AtCtpA, GST-AtCtpB, and GST-AtCtpC were unable to process pD1 in this *in vitro* assay under different pH conditions even when the reaction time was extended to 60 min (Figures 5C,D). This demonstrates that neither AtCtpB nor AtCtpC is able to cleave the pD1 C-terminal extension.

To test which Ctp domain is required for pD1 processing activity, we conducted a domain-swapping assay between AtCtpA and AtCtpB. The three domains of AtCtpA were replaced with their counterparts from AtCtpB, respectively, generating three new recombinants designated as AtCtpR1 (switching domain A), AtCtpR2 (switching domain B), and AtCtpR3 (switching domain C) (Supplementary Figure 6B). These proteins were used in the Ctp protease assay with synthetic recombinant pD1 as substrates as described above. None of the recombinant Ctp proteins could convert pD1 into mD1 (Supplementary Figure 6C). Therefore, although AtCtpB is similar to AtCtpA topologically, all three domains in AtCtpB failed to catalyze pD1 processing.

### CtpB and CtpC From Other Model Photosynthetic Organisms Cannot Process pD1 in Arabidopsis *in vivo* or *in vitro*

**FIGURE 8:**
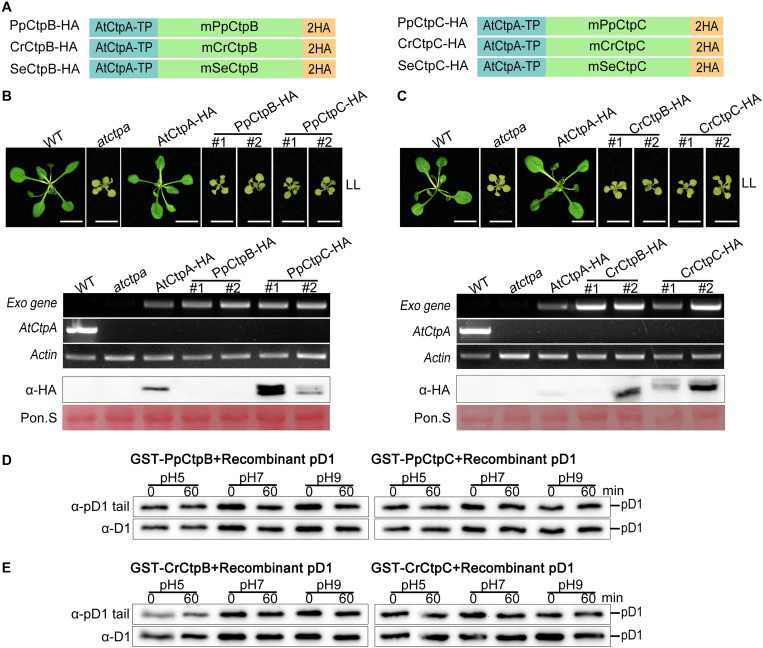
Proteolytic activity of CtpB and CtpC from lower photosynthetic organisms for pD1 processing. **(A)** Schematic diagrams of *PpCtpB-HA* or *PpCtpC-HA* (from *P. patens*), *CrCtpB-HA* or *CrCtpC-HA* (from *C. reinhardtii*), and *SeCtpB-HA* or *SeCtpC-HA* (from *S. elongatus*). *AtCtpA-TP* is the N-terminal chloroplast transit peptide of AtCtpA; mPpCtpB or C, mCrCtpB or C, and mSeCtpB or C are the mature proteins; 2HA is a double hemagglutinin tag. **(B,C)** Ectopic expression of *PpCtpB-HA* or *PpCtpC-HA* and *CrCtpB-HA* or *CrCtpC-HA* driven by the CaMV 35S promoter in *atctpa* was analyzed as described in the legend of Figure 7. **(D,E)** *In vitro* protease activity of PpCtpB or C and CrCtpB or C were assayed with recombinant AtpD1 as substrate as described in the legend of Figure 4.

CtpB and CtpC of three model organisms were introduced and expressed in the *atctpa* mutant in the same way as described above (Figure 8A). However, we were not able to obtain positive T1 plants transformed with *SeCtpB-HA* or *SeCtpC-HA* (screened > 10,000 T1 seeds from several transformation experiments). We examined the transgenic *atctpa* plants carrying *PpCtpB-HA*, *PpCtpC-HA*, *CrCtpB-HA*, *or CrCtpC-HA*. Similar to *AtCtpB-HA* and *AtCtpC-HA*, ectopic expression of *PpCtpB-HA*, *PpCtpC-HA*, *CrCtpB-HA*, or *CrCtpC-HA* in *atctpa* did not rescue *atctpa* (Figures 8B,C).

Moreover, we expressed and purified GST-PpCtpB or C and GST-CrCtpB or C in *E. coli* cells (Supplementary Figure 7) and used these proteins for the *in vitro* Ctp activity test with recombinant pD1 substrate. None of them cleaved the C-terminal end of the recombinant pD1 substrate after a 60 min incubation at pH 5.0, 7.0, and 9.0 (Figures 8D,E). These results were consistent with their evolutionary data indicating that eukaryotic CtpB and CtpC may have diverged from CtpA long before photosynthetic organisms emerged, and thus did not evolve the pD1 processing function. Because SeCtpB or C may have a detrimental effect on *E. coli* and Arabidopsis, we could not obtain recombinant proteins and transgenic plants of SeCtpB or C to perform these *in vivo* and *in vitro* tests.

In addition, GST-PpCtpB or C and GST-CrCtpB or C were unable to process two prokaryotic and eukaryotic pD1 substrates (Figures 6C, 8). Once again, we conclude that only CtpA is responsible for the C-terminal maturation of D1 while CtpB and CtpC are not involved in this process.

## Discussion and Conclusion

Unlike other PSII subunits encoded by the plastid genome, D1 is initially synthesized as a precursor (pD1) with a short extension at its C-terminus. The pD1 precursor needs to be processed to generate functional D1 protein (mD1). The C-terminal peptidase (Ctp) is responsible for D1 maturation by cleaving the pD1 C-terminal extension. Usually, the genomes of most oxygenic photosynthetic organisms encode three Ctp homologs named CtpA, CtpB, and CtpC (Satoh and Yamamoto, 2007). In this study, we explored the roles of these three Ctp homologs in D1 maturation in Arabidopsis and uncovered the evolutionary and functional divergence of the Ctp family in oxygenic photosynthetic organisms.

### AtCtpA Is the Only Enzyme Responsible for D1 Maturation in Arabidopsis

Analysis of Arabidopsis *Ctp* deficient mutants showed that the *atctpa* mutant displayed a yellowish and arrested growth phenotype and retained its D1 protein in the pD1 form (Figure 2). In contrast, single and double mutants of *atctpb* and *atctpc* all grew as WT under the tested conditions and did not show a defect in pD1 processing and PSII SC assembly (Figure 2, Supplementary Figure 3). AtCtpB or AtCtpC gene driven by 35S promoter did not rescue the *atctpa* phenotype (Figure 7), and AtCtpB or AtCtpC were not able to cleave the pD1 C-terminal extension *in vitro* (Figure 5). These results, combined with previous studies on AtCtpA (Che et al., 2013) and AtCtpB (Yin et al., 2008) indicate that neither AtCtpB nor AtCtpC functions in D1 maturation, a process that is uniquely catalyzed by AtCtpA.

It is not surprising that AtCtpB and AtCtpC do not have the capacity to process the pD1 C-terminal extension because they are only distantly related to AtCtpA and share a relative low sequence identity (around 36%) (Supplementary Figure 6). The domain swapping experiments showed that replacement of any of three domains in AtCtpA with counterparts from AtCtpB compromises its proteolytic activity for processing pD1 (Supplementary Figure 6). Thus, it is possible that structural domains in AtCtpB cannot adopt the proper conformation required for pD1 processing. However, the whole tertiary structures are quite similar in the three Ctps implying that the substrate specificity is restricted to AtCtpA, not to AtCtpB or AtCtpC.

All Arabidopsis *Ctp* genes are expressed in leaf tissues (Figure 2A) suggesting that AtCtpB and AtCtpC have some physiological roles in chloroplasts. AtCtpB was shown to be involved in PSII repair (Yin et al., 2008). However, the function of AtCtpC is still unknown. We could not find any obvious abnormal phenotype in *atctpc* mutant plants suggesting that the deficiency in *atctpc* is very subtle, and extra fine analysis should be performed to dissect the function of AtCtpC.

### CtpA Acquired the Activity to Process pD1 During Evolution of Oxygenic Photosynthetic Organisms

Together with previous studies, we showed that CtpA is the only enzyme responsible for D1 maturation as no other protease can substitute for its function in *Synechocystis sp.* PCC 6803, *S. obliquus*, and Arabidopsis (Anbudurai et al., 1994; Trost et al., 1997; Che et al., 2013). Considering the high conservation of the photosynthetic apparatus, CtpA emerges as the only enzyme capable to cleave the C-terminal extension of pD1 in oxygenic photosynthetic organisms.

Ectopic expression of SeCtpA, CrCtpA, and PpCtpA in the *atctpa* mutant can rescue the lethal phenotype of the *atctpa* mutant indicating that the CtpA function is conserved in oxygenic photosynthetic organisms. *In vitro* enzyme assays showed that CtpA can process pD1 from either Arabidopsis or cyanobacteria (Figures 4–6). In contrast, ectopic expression of CtpB or C from *C. reinhardtii* and *P. patens* in *atctpa* failed to rescue plant growth. These CtpB or C proteins also could not cleave the pD1 tail from Arabidopsis or cyanobacteria *in vitro* (Figures 5, 6, 8), confirming that CtpB or C lack the pD1 cleavage activity.

The phylogenetic analysis showed that CtpAs from prokaryotes and eukaryotes belong to the same evolutionary lineage and share a recent common ancestor, while CtpB and CtpC belong to distinct evolutionary lineages (Figure 1 and Supplementary Figure 1). Notably, eukaryotic CtpB and CtpC have diverged earlier even before cyanobacteria emerged, and they are more closely to *E. coli* Tsp, suggesting that eukaryotic CtpB and CtpC likely originated from the hosts in an endosymbiotic event or were acquired via horizontal gene transfer (HGT) from ancient bacteria. Recent analyses reveal that HGT from bacteria to eukaryotes often occurred during the evolution of eukaryotes (Schonknecht et al., 2013; Husnik and McCutcheon, 2018). The divergent evolutionary relationship between CtpA and CtpB or C strongly suggests that these proteins have different functions.

Based on the expression of Ctps from three representative model organisms in the *atctpa* mutant, *in vitro* enzyme assays with pD1 substrate from Arabidopsis and cyanobacteria, and the phylogenetic analysis, we conclude that CtpA is exclusively responsible for D1 maturation in oxygenic photosynthetic organisms, and the other two homologs, CtpB and CtpC have a different role. Previous report showed that low level of pD1 processing can occur in absence of CtpA in Cyanobacteria (Komenda et al., 2007), but we have now found that CtpB and CtpC are not involved in processing pD1, therefore the remaining processing might be performed by another protease.

### The Relationship Between CtpA and Its pD1 Substrate

CtpA enzyme activity displayed a pH dependence with different pD1 substrates (Yamamoto et al., 2001; Figures 4–6). In particular, CtpA had an optimal activity at pH 8.0 or 9.0 for cleaving a synthetic pD1 C-terminal peptide. In contrast, with native membrane-embedded pD1, it exhibited the highest activity at pH 5.0–6.0, close to the expected pH value of the thylakoid lumen (Takizawa et al., 2007; Tikhonov et al., 2008). To process the pD1 protein embedded in the thylakoid membrane, CtpA needs to associate with the thylakoid membrane to contact its substrate. A structure analysis showed that five conserved residues in the A domain of SoCtpA may serve as the membrane recognition site (Liao et al., 2000). The low pH environment will confer positive charges to these residues, which would facilitate the interaction of CtpA with the negatively charged membrane and thereby allow for access to pD1. Some factors could be involved in regulating CtpA activity, for instance, PratA in cyanobacteria (Klinkert et al., 2004), and LPA19 in Arabidopsis (Wei et al., 2010).

SeCtpA, CrCtpA, and PpCtpA are capable of cleaving the C-terminal extension of pD1 in Arabidopsis (Figures 3, 4). When pD1 from Arabidopsis was used as the substrate, the *in vivo* and *in vitro* results showed that the order of Ctp enzyme activity is AtCtpA > PpCtpA > CrCtpA > SeCtpA consistent with the convergent evolutionary relationship between CtpA and its substrate. However, when a synthetic Se-pD1 was used as substrate, the order of enzyme activity is still AtCtpA > PpCtpA > CrCtpA. Therefore, it is likely that CtpAs from oxygenic photosynthetic organisms became more efficient along with the evolutionary lineage irrespective of the source of pD1.

The sequence upstream of the cleavage site of pD1 is extremely conserved while the C-terminal extension of pD1 shows a large diversity both in terms of length and sequence (Figure 6A). The pD1 C-terminal extension of cyanobacteria is 16 aa long and higher plants usually carry a 9 aa long C-terminal extension of pD1. This suggests that the pD1 C-terminal extension became shorter during evolution. The fact that CtpA from these four different species could process pD1 from both Arabidopsis and cyanobacteria (Figures 3–6) indicates that the conserved N-side sequence before the cleavage site is more important than the C-terminal extension *per se* for CtpA. However, we also noticed that AtCtpA and PpCtpA were more efficient with the synthetic Arabidopsis pD1 than with the synthetic cyanobacterial pD1 (Figures 4–6) suggesting that the C-terminal extension does affect the CtpA enzyme activity, and CtpA prefers a short C-terminal extension rather than a long one. We cannot exclude the possibility that two-step cleavage occurred in longer C-terminal extension can also slow down the rate of cleavage (Inagaki et al., 2001b).

### An Evolutionary Scenario of Oxygenic Photosynthesis Based on CtpA

Current photosynthetic organisms can be classified into two groups: non-oxygenic or oxygenic photosynthetic organisms. Oxygenic photosynthetic organisms have a D1/D2 type PSII, while non-oxygenic organisms harbor an L/M type PSII. Both L/M type PSII and D1/D2 type PSII originated from a common ancient non-oxygenic PSII (Cardona, 2015; Cardona et al., 2018). The original D1/D2 type PSII should be a non-oxygenic PSII due to the presence of the pD1 C-terminal extension which would block the association of the MnCa-Cluster. Here, we propose that one event among others which initiated the emergency of oxygenic photosynthesis was the evolution of a Tsp-like peptidase into CtpA that specifically cuts off the C-terminus of ancient pD1. This would allow the docking of the MnCa-Cluster to the D1 protein and lead to the formation of the initial oxygen evolving D1/D2 type PSII. Thereafter, as aqueous oxygenic photosynthetic organisms evolved into land plants, photo-damage under excessive light became a major threat. To cope with this new challenge, photosynthetic organisms developed the photo-damage repair mechanism, which consequently required more efficient D1 turnover. Accordingly, this may have led to a higher efficiency of CtpA and, possibly, the shortening of the C-terminal extension during evolution.

## Data Availability Statement

The original contributions presented in the study are included in the article/Supplementary Material, further inquiries can be directed to the corresponding author.

## Author Contributions

AF, MX, and YW conceived the research plans. WC, CL, ZC, WL, HS, HC, WF, CW, TH, YL, and YS performed the experiments. AF, MX, FW, and WC analyzed the data. WC, AF, and MX wrote the manuscript with contributions from all authors. AF agrees to serve as the author responsible for contact and ensure communication.

## Conflict of Interest

The authors declare that the research was conducted in the absence of any commercial or financial relationships that could be construed as a potential conflict of interest.

## Footnotes

1 https://www.ncbi.nlm.nih.gov/

2 https://phytozome.jgi.doe.gov/pz/portal.html

3 https://www.rcsb.org/structure/1fcf
