# Supplementary figures and images for: Diverged Early From CtpB and CtpC, CtpA Has Evolved to Process D1 Precursor in Oxygenic Photosynthetic Organisms
Source: Front Plant Sci. 2021 Apr 30;12:676036. doi: 10.3389/fpls.2021.676036 (PMC8121967; doi:10.3389/fpls.2021.676036)

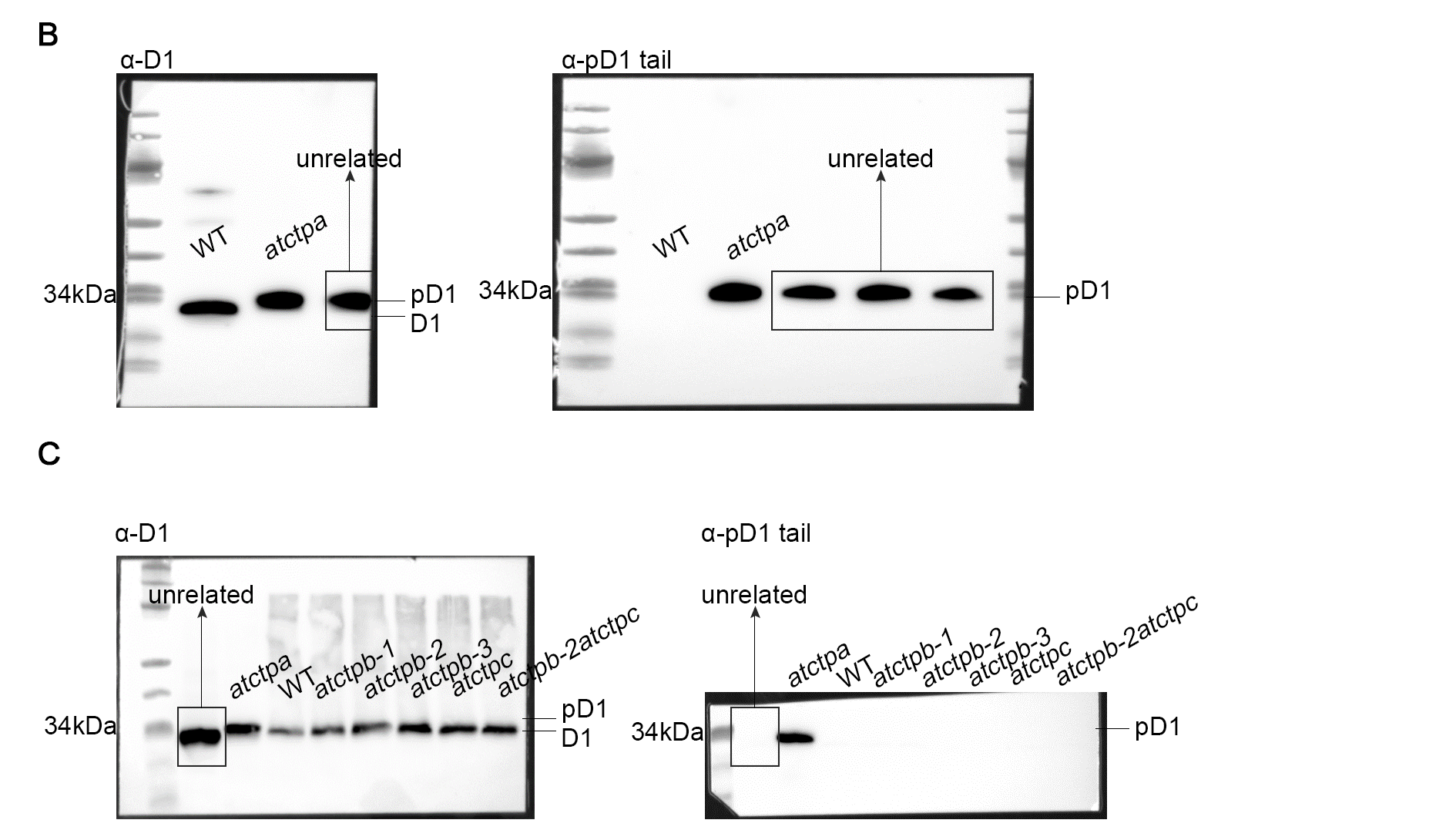

Supplement: Supplementary file 1 [file Image_1.TIF]

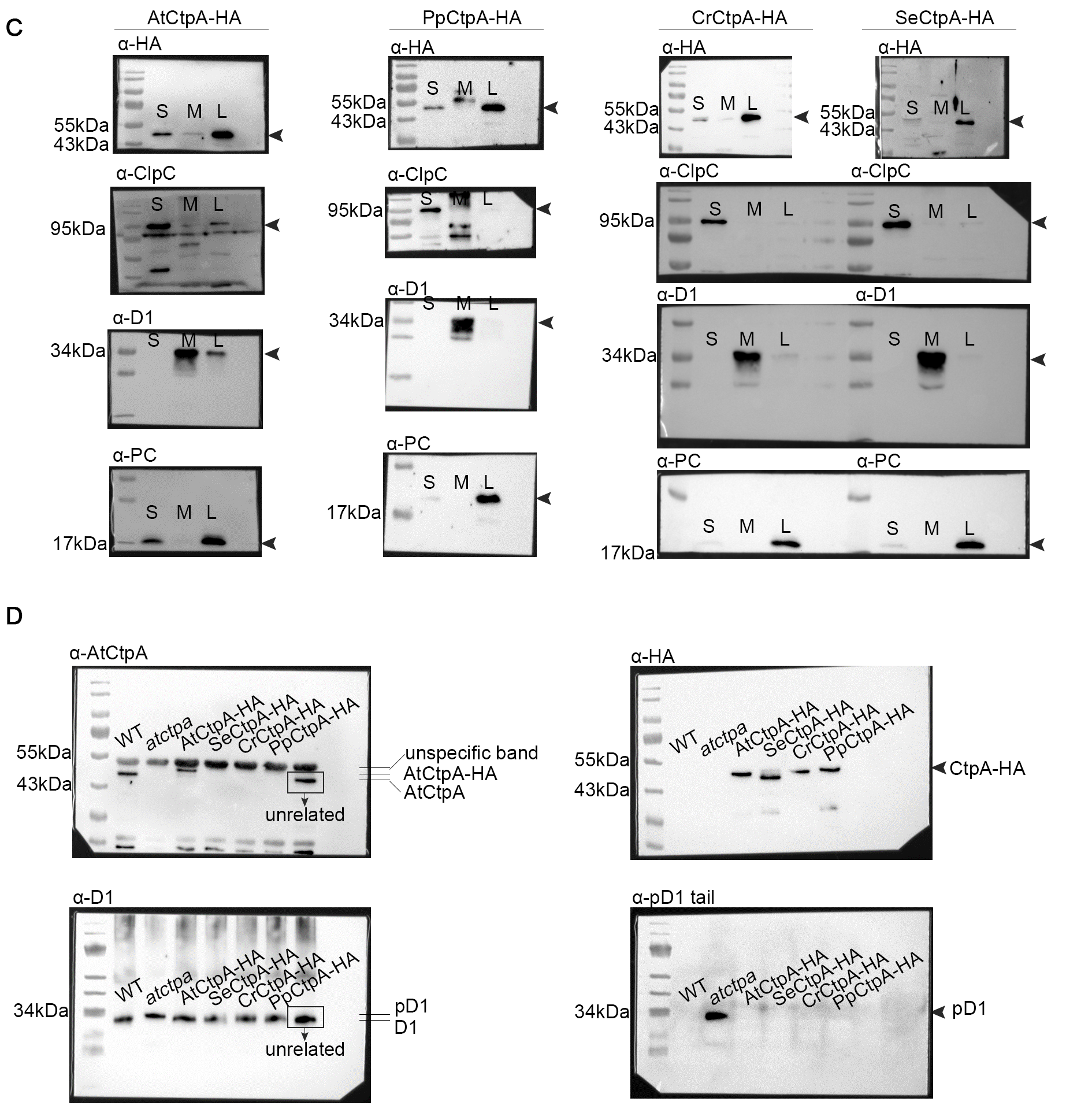

Supplement: Supplementary file 2 [file Image_2.TIF]

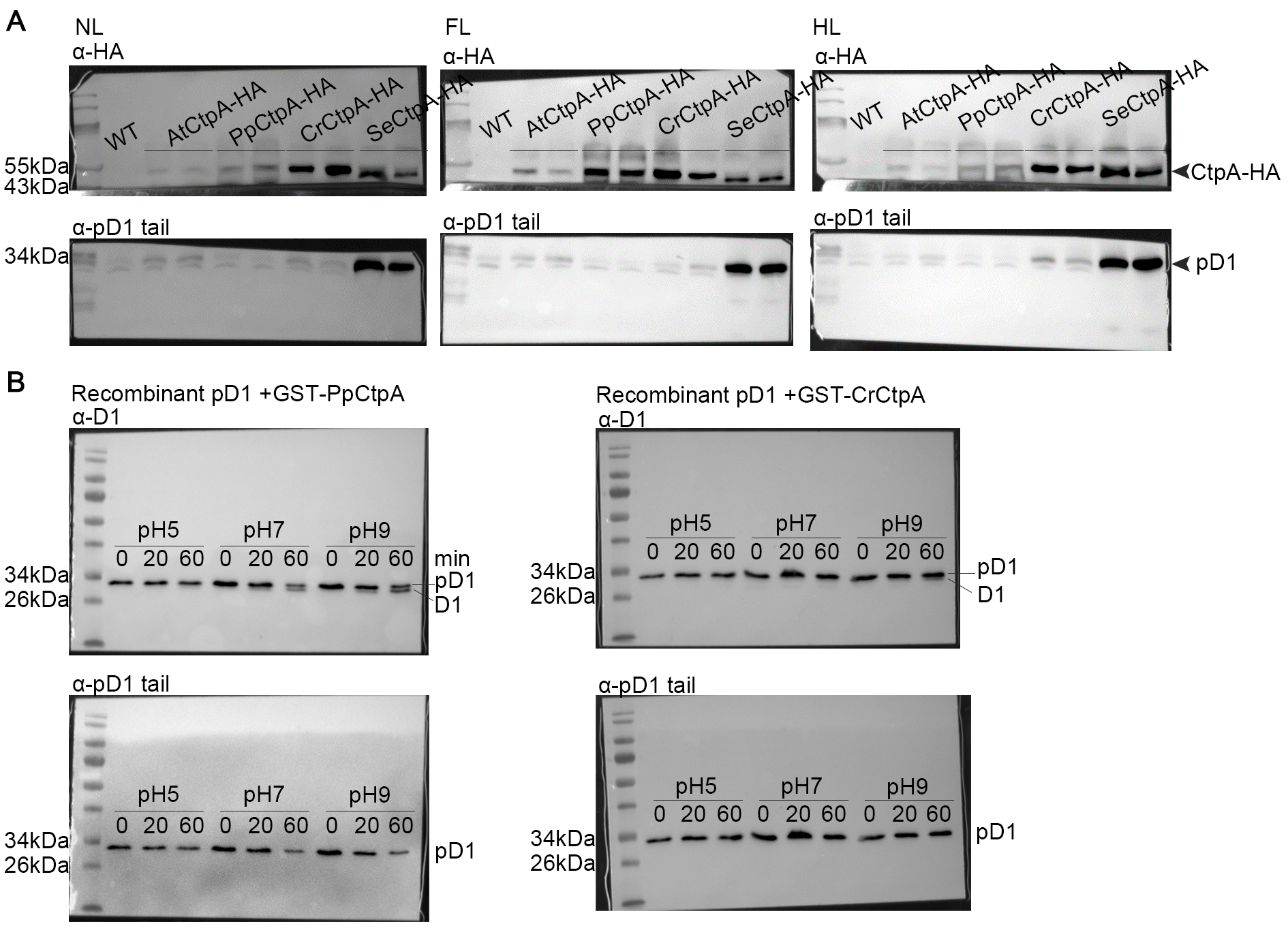

Supplement: Supplementary file 3 [file Image_3.TIF]

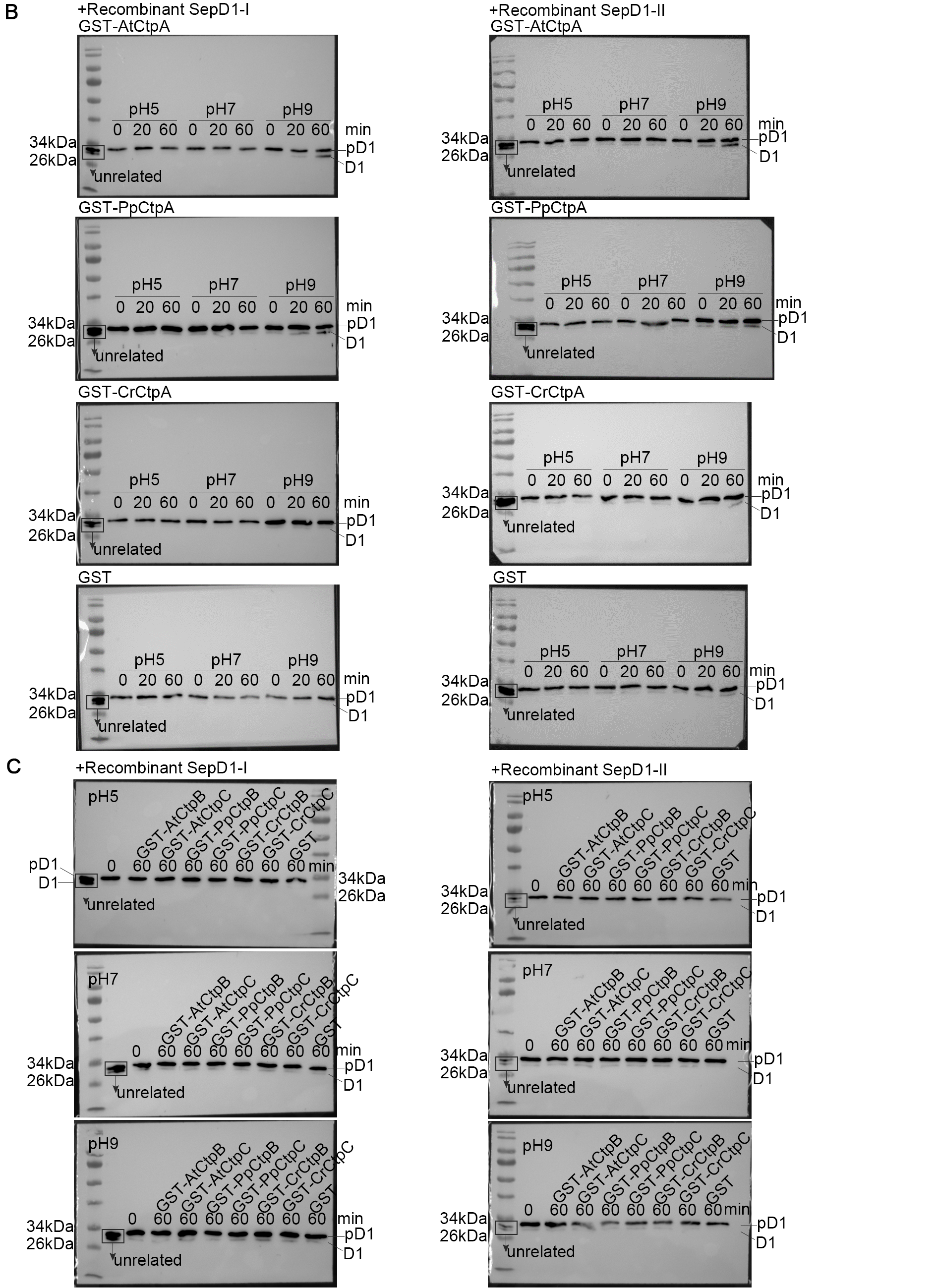

Supplement: Supplementary file 4 [file Image_4.TIF]

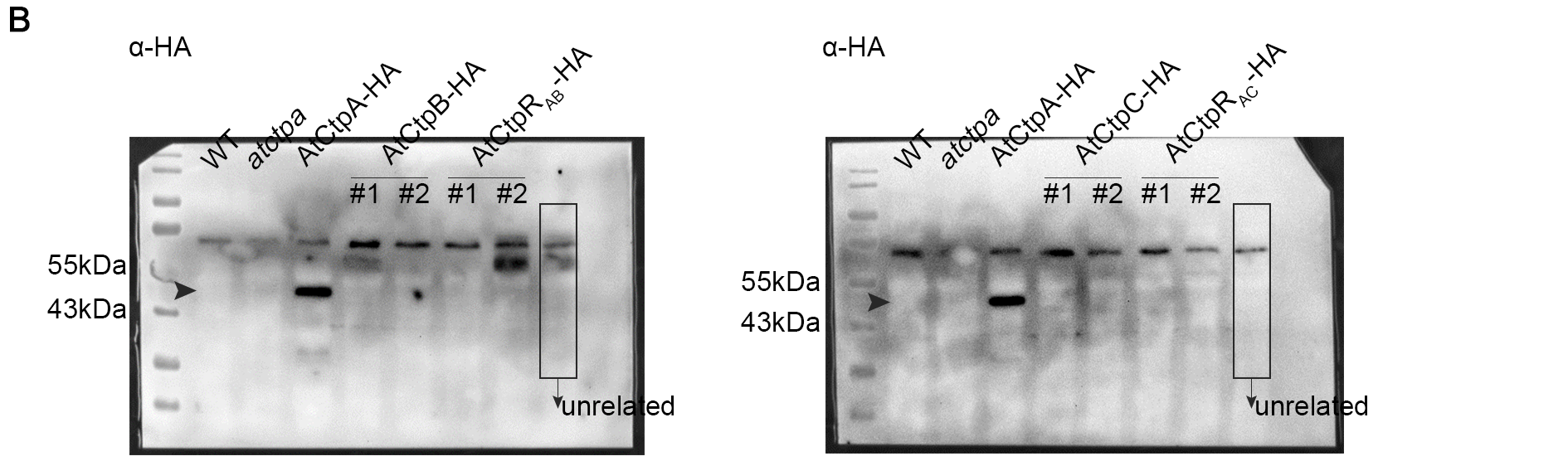

Supplement: Supplementary file 5 [file Image_5.TIF]

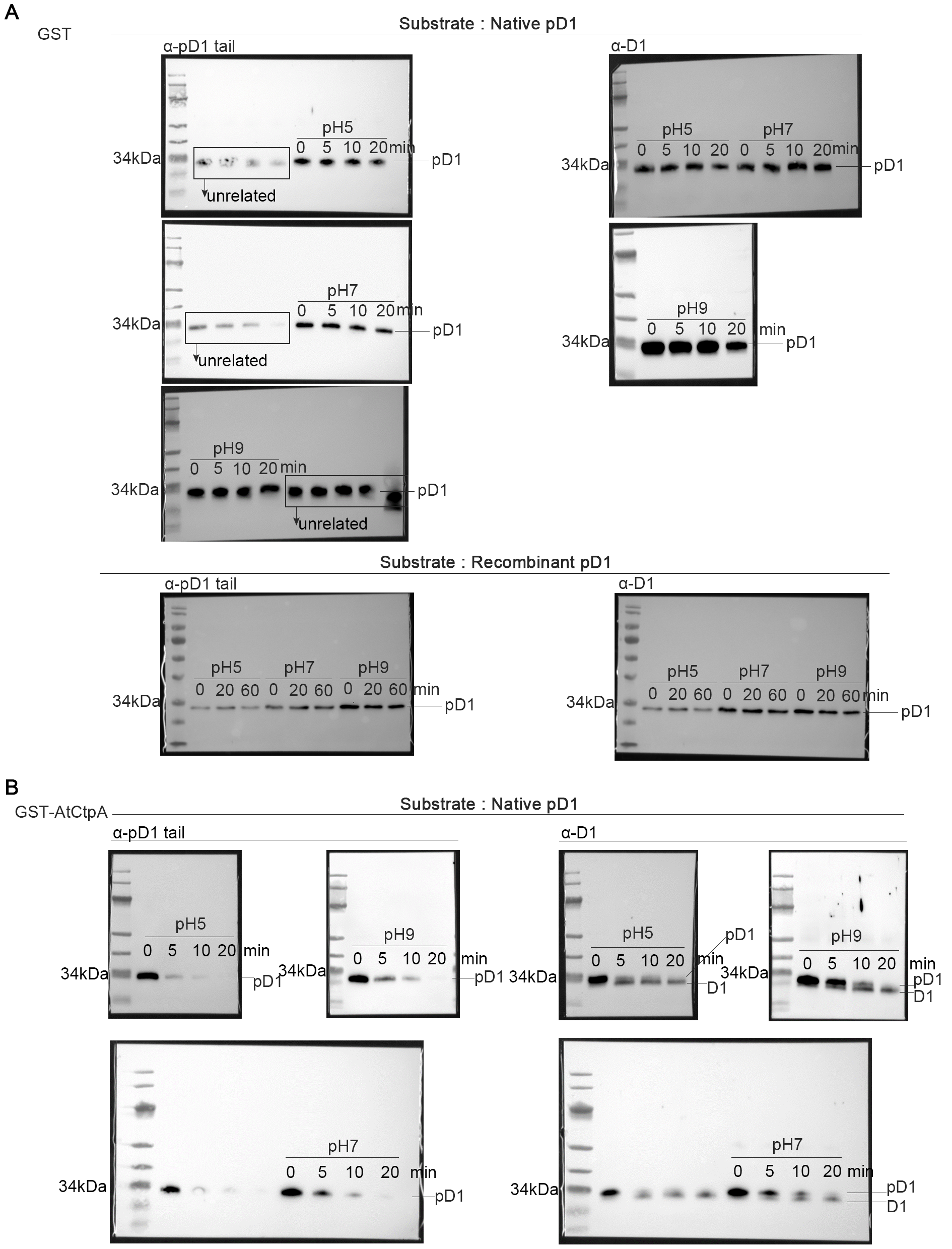

Supplement: Supplementary file 6 [file Image_6.TIF]

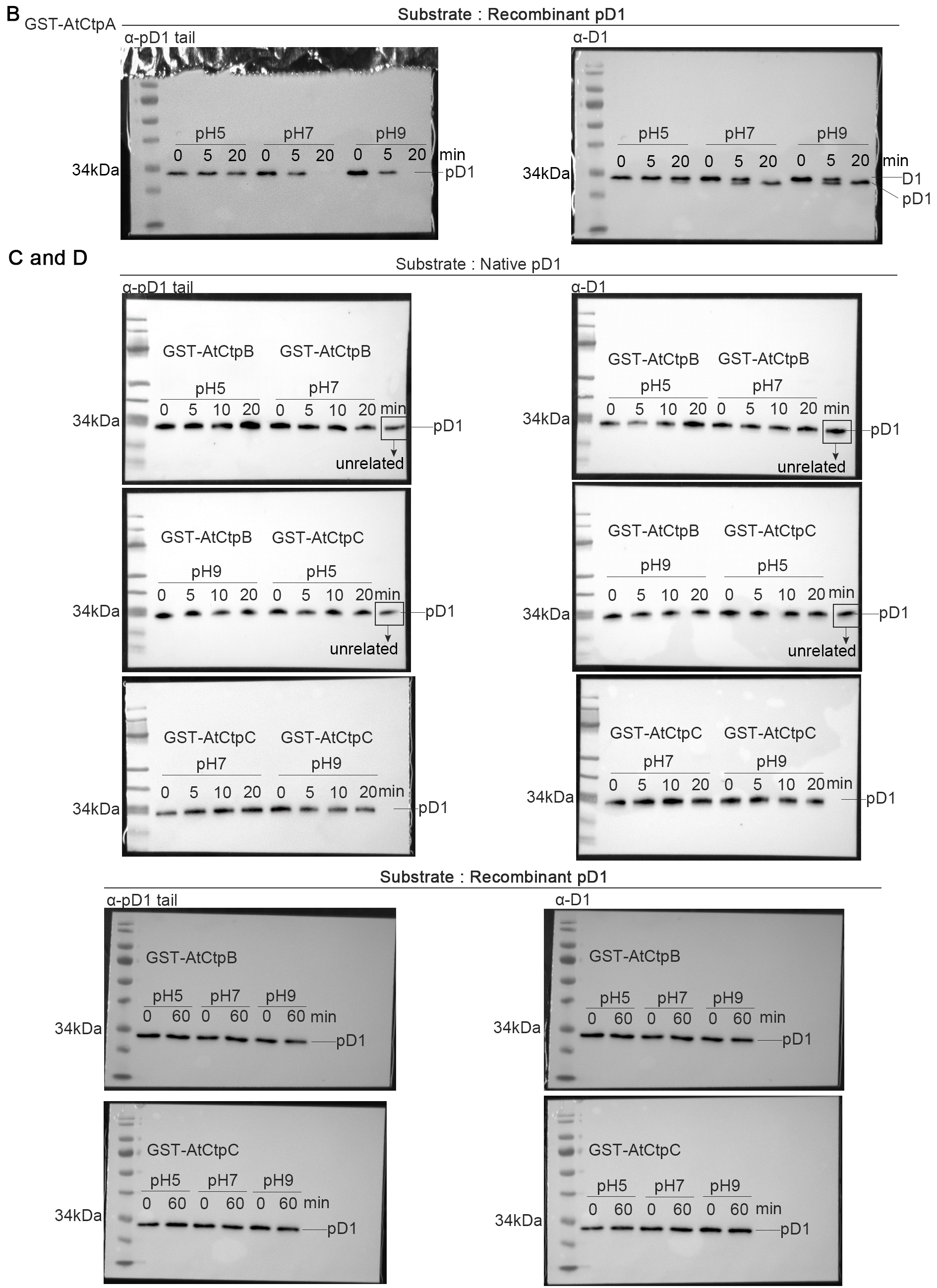

Supplement: Supplementary file 7 [file Image_7.TIF]

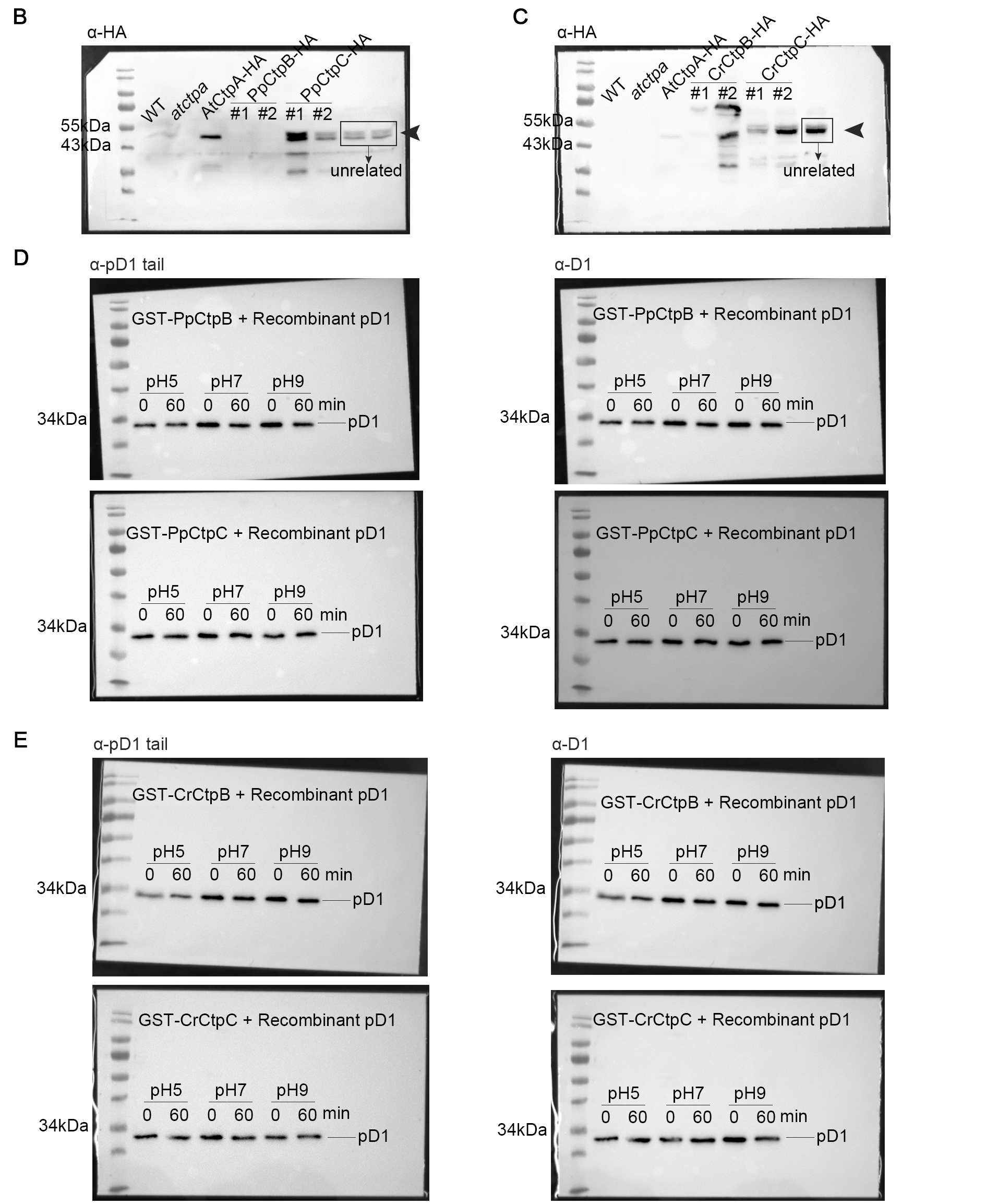

Supplement: Supplementary file 8 [file Image_8.TIF]

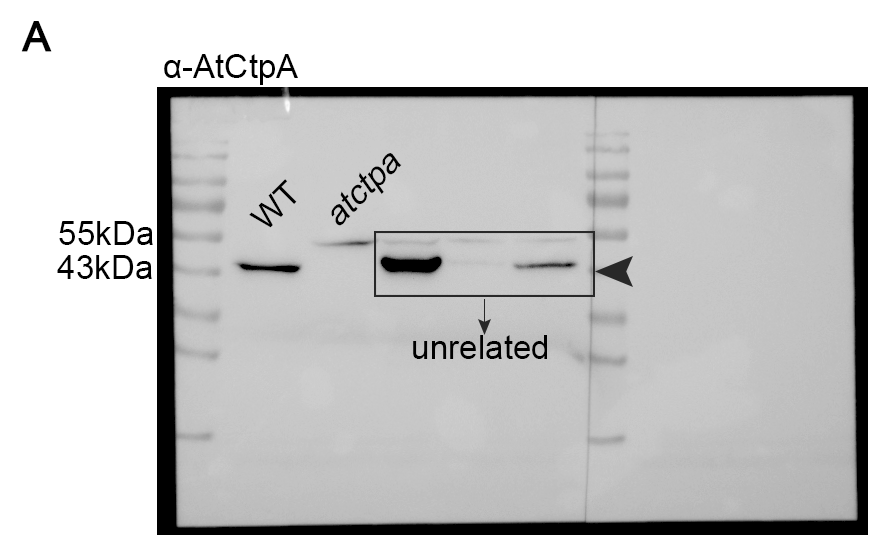

Supplement: Supplementary file 9 [file Image_9.TIF]

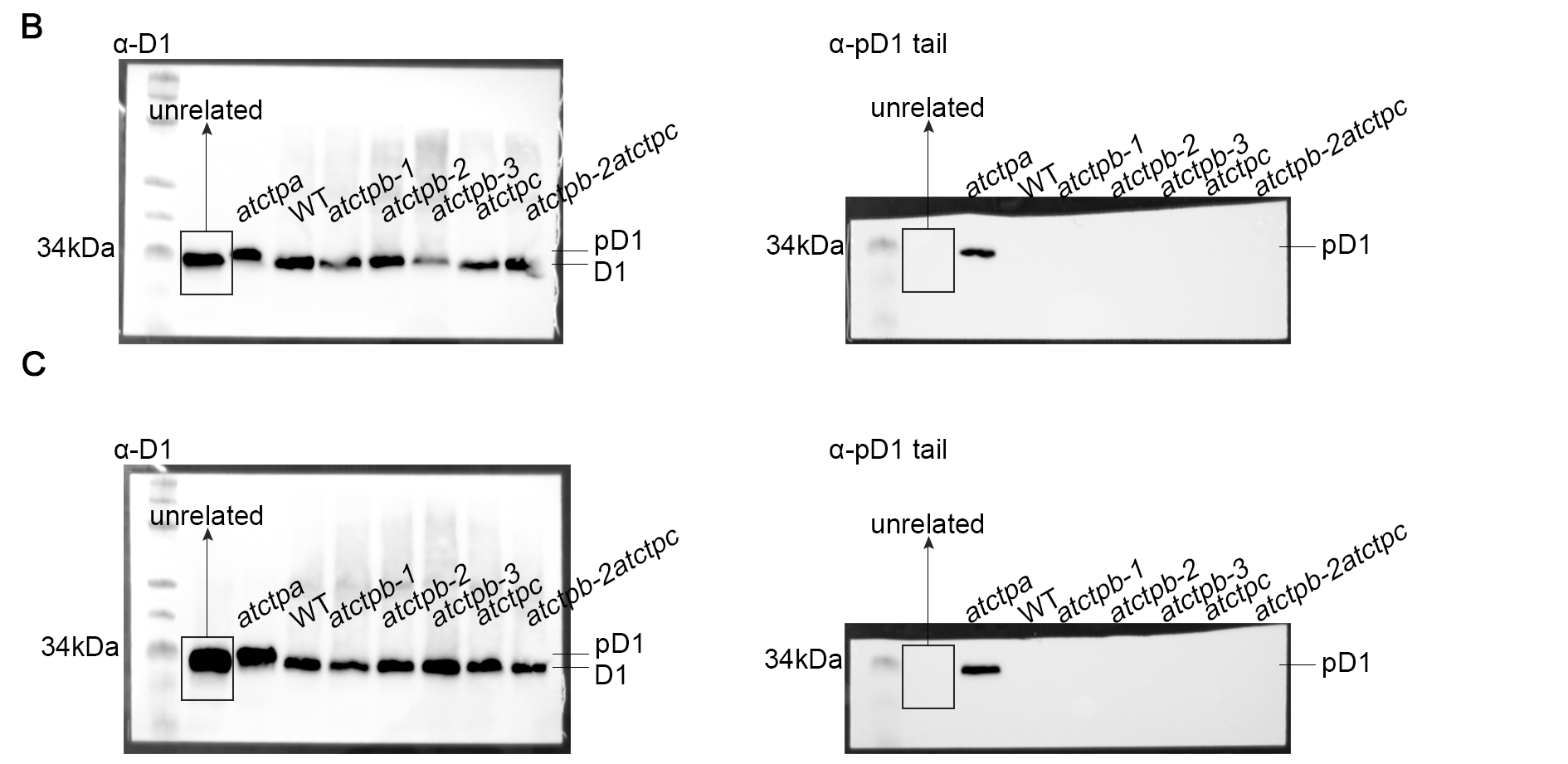

Supplement: Supplementary file 10 [file Image_10.TIF]

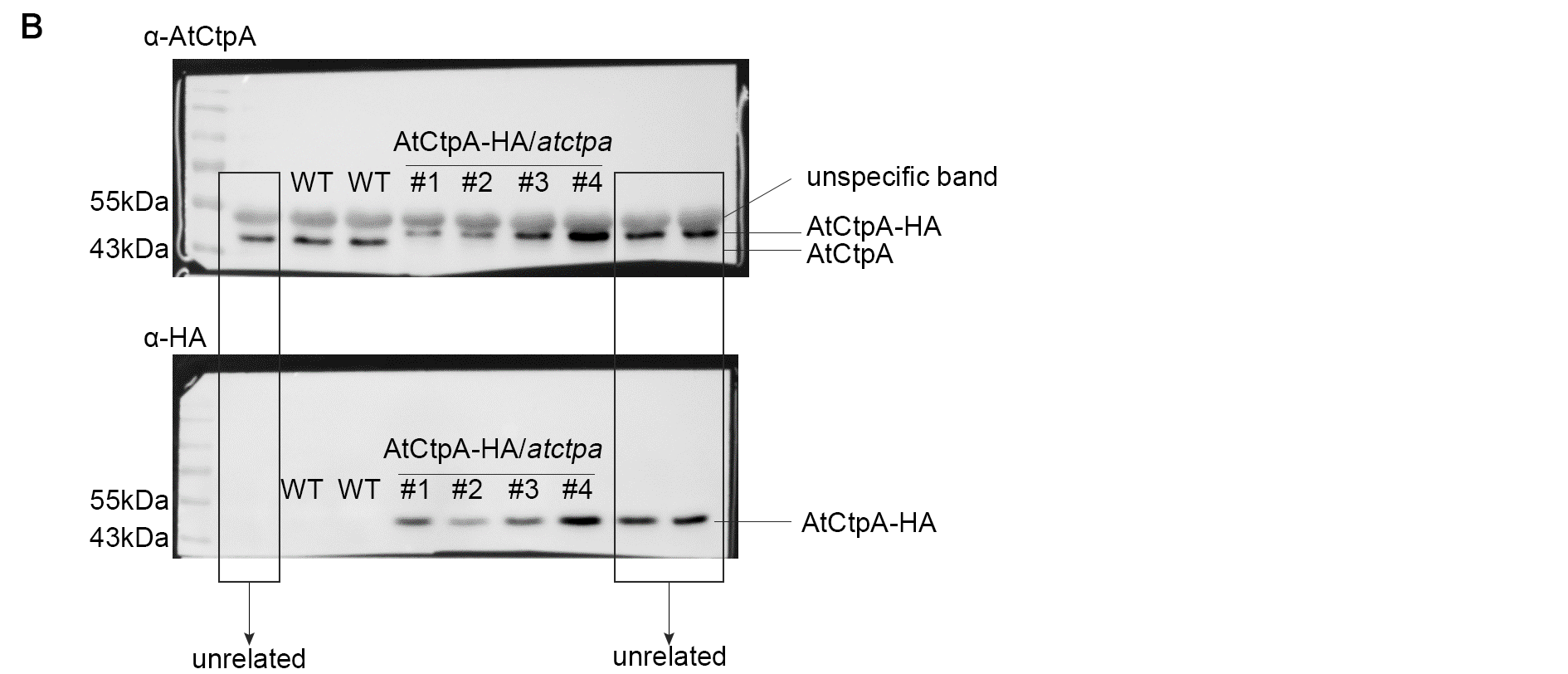

Supplement: Supplementary file 11 [file Image_11.TIF]

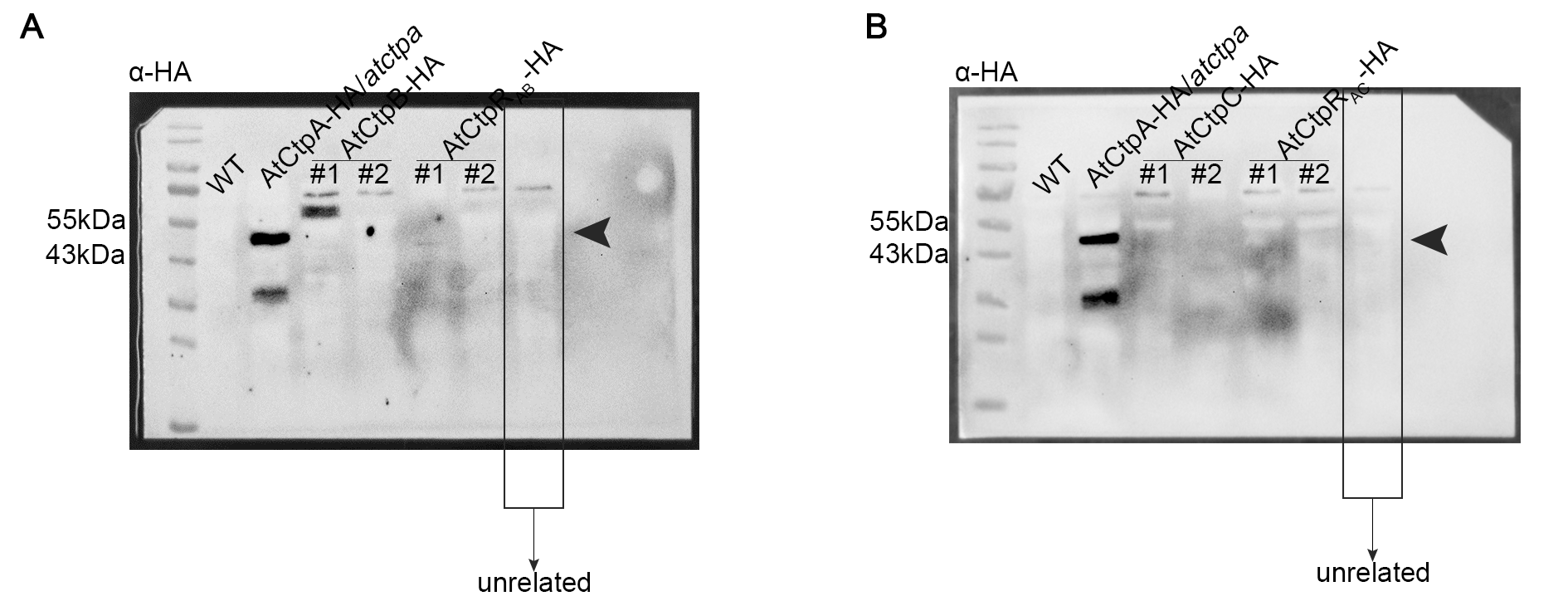

Supplement: Supplementary file 12 [file Image_12.TIF]

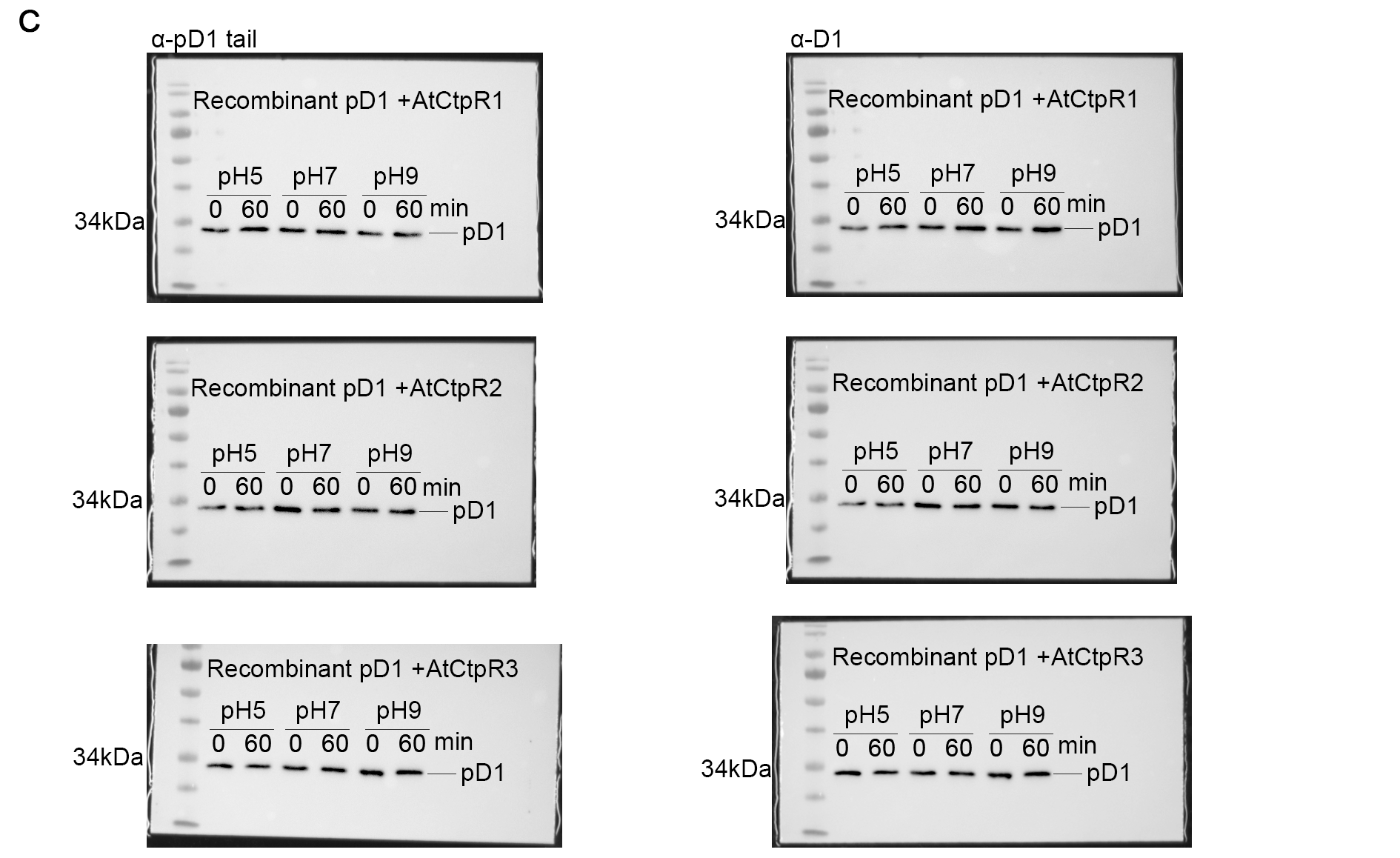

Supplement: Supplementary file 13 [file Image_13.TIF]
