# Supplementary material for: Diverged Early From CtpB and CtpC, CtpA Has Evolved to Process D1 Precursor in Oxygenic Photosynthetic Organisms
Source: Front Plant Sci. 2021 Apr 30;12:676036. doi: 10.3389/fpls.2021.676036 (PMC8121967; doi:10.3389/fpls.2021.676036)
Supplement: Supplementary file 14 [file Data_Sheet_1.pdf]

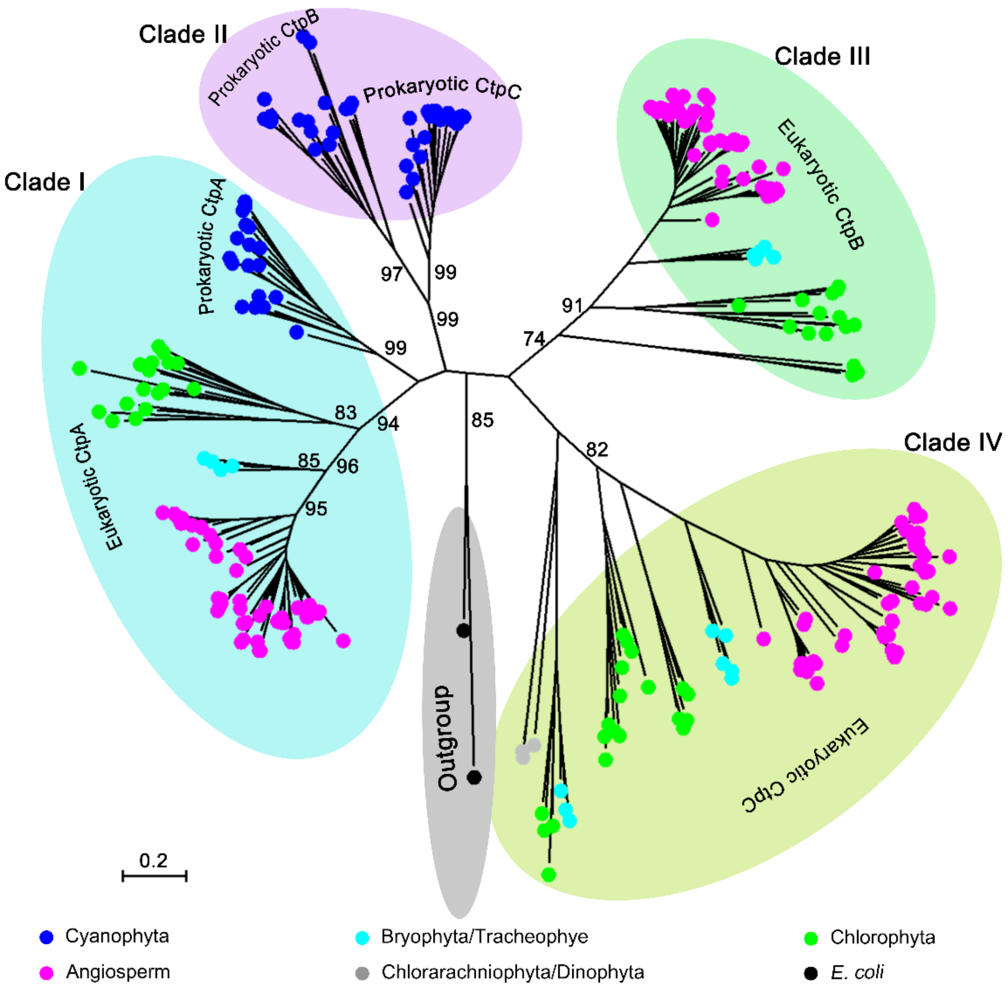

Supplementary Figure S1. Phylogenetic tree constructed by Maximum-Likelihood method.

To make the relationships of Ctp clades in Figure 1 more trustworthy, 286 Ctp proteins analyzed in figure 1 were also used to construct phylogenetic tree by MAGA6 using the method of Maximum-Likelihood, with the bootstrapping value set at 500 replications. The new tree displays the same topology as in figure 1.

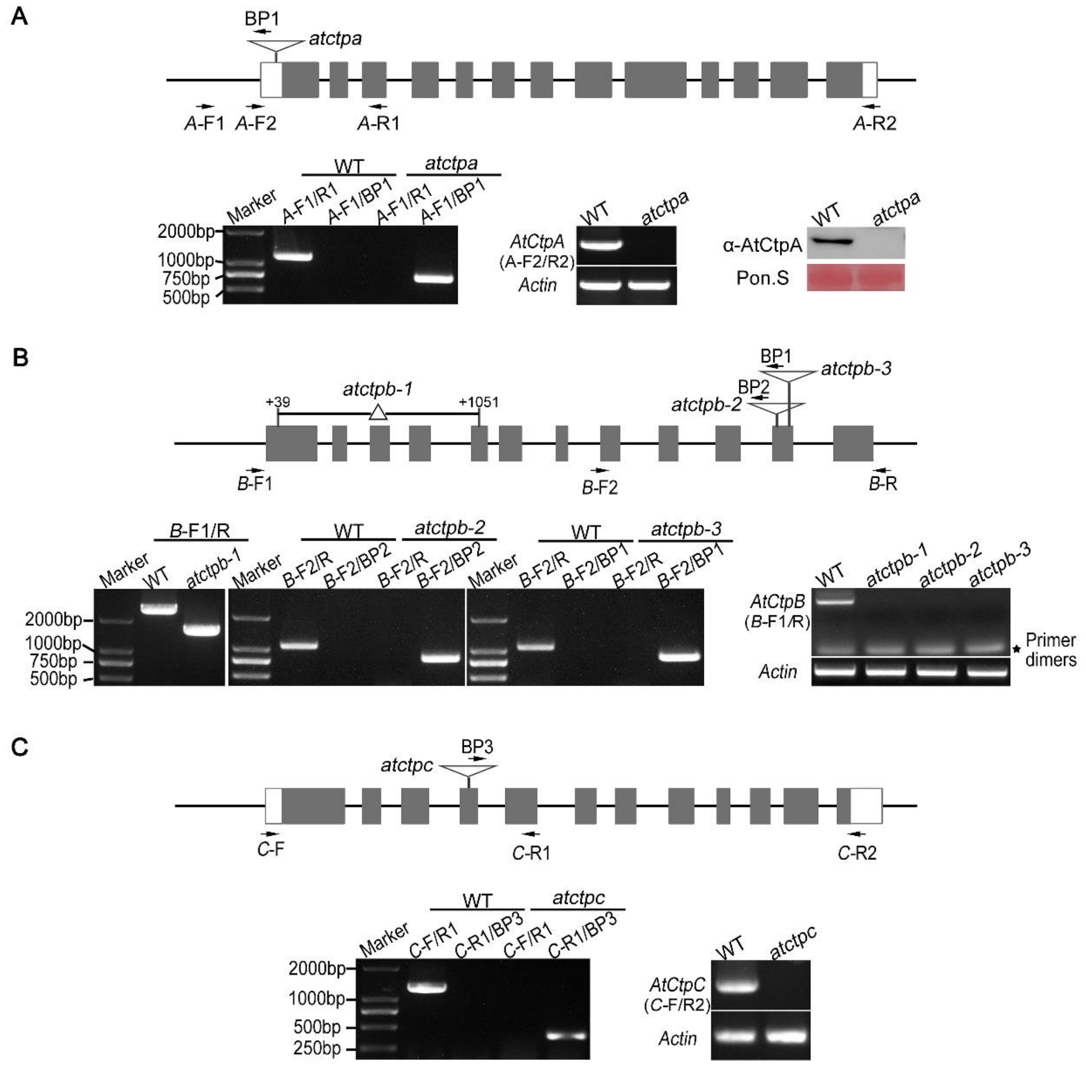

Supplementary Figure S2. Molecular characterizations of *atctpa* (A), *atctpb* (B), and *atctpc* (C) mutants.

Upper panels in (A), (B), and (C) are schematic representations of different alleles of *atctpa*, *atctpb*, and *atctpc*. White boxes, the 5'- and 3'-UTR of genes; grey boxes, exons; black lines through boxes, the flanking regions and introns of genes; inverted triangles, T-DNA insertions; Δ, DNA deletions; horizontal arrows, PCR primers used for characterizing mutants. Molecular characterizations of mutants were placed below the schematics, including genotyping by PCR (left) and transcription level analyses by RT-PCR (right in (B) and (C) or middle in (A)), of which the forward primer (*A-F2*) for analysis of *AtCtpA* transcription level is located in the 5'-UTR region before the T-DNA insertion site. For *atctpa* mutant, AtCtpA protein level was further analyzed by immunoblot against α-AtCtpA antibody (right). The filters stained with Ponceau S (Pon. S) were used as loading controls.

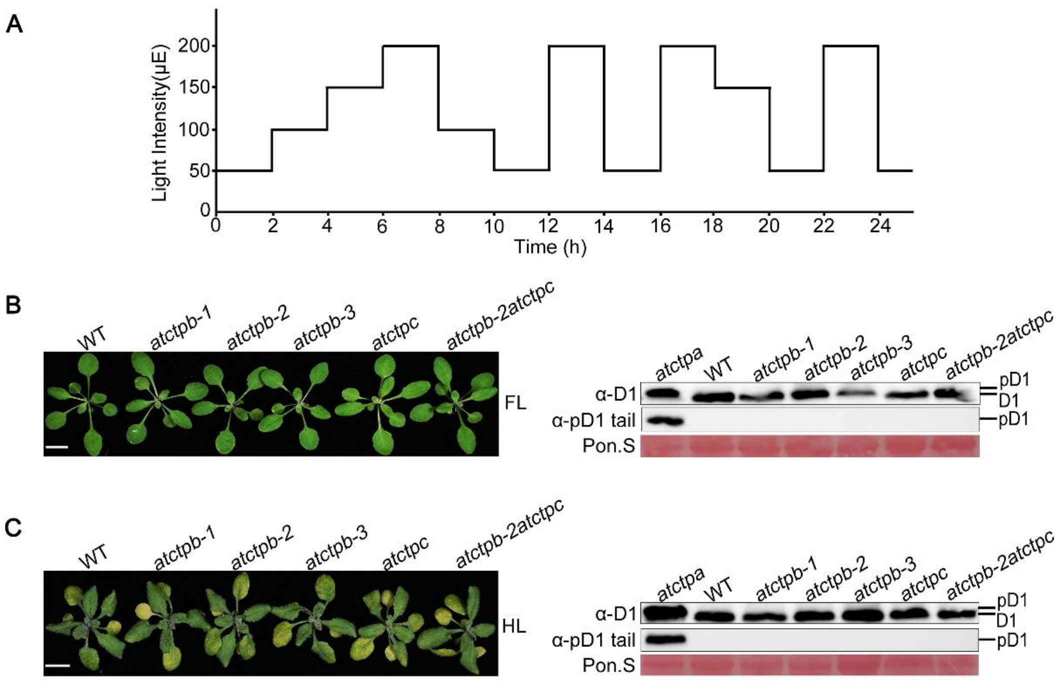

Supplementary Figure S3. Analyses of *atctpb* and *atctpc* mutants under FL and HL light conditions.

(A) Schematic diagram of light intensity changes during a 24-hour period (Fluctuating light, FL).

(B) and **(C)** Characterizations of the phenotypes (left) and pD1 processing statuses (right) of *atctpb* and *atctpc* mutants under FL and HL conditions. The plants were gown in soil for three weeks as the following continuous light conditions: FL, under normal light (NL, 80 μmol/s/m^2) for one week and then under FL for two weeks; high light (HL, 350 μmol/s/m^2), under NL for two weeks and then under HL for one week. Scale bars denote 1 cm. After photographed, the pD1 processing statuses in plants were analyzed by immunoblot against α-D1 (for D1 and pD1) and α-pD1 tail (for pD1). The filters stained with Ponceau S (Pon. S) were used as loading controls.

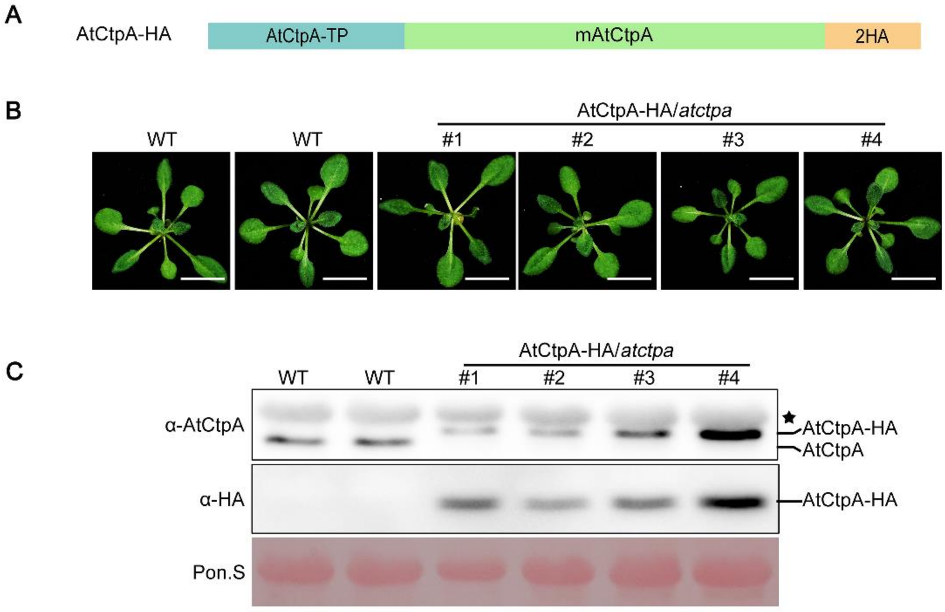

Supplementary Figure S4. Complementation of *atctpa* mutant phenotypes with *AtCtpA*.

(A) Schematic diagram of AtCtpA-HA for the complementation analysis. AtCtpA-TP is the chloroplastic transit peptide, mAtCtpA is the mature peptide; the 2HA indicates a double hemagglutinin tag.

(B) The phenotypes of transgenic *atctpa* plants carrying CaMV 35S promoter-driven *AtCtpA-HA*. The plants were grown on 1/2 MS medium with 1% sucrose under continuous low light (LL, 25 μmol/s/m^2) for three weeks. AtCtpA-HA #1-4 indicate different independent transgenic lines. Scale bars represent 1cm.

(C) The AtCtpA proteins levels of the plants in (B) were analyzed by immunoblot with α-AtCtpA and α-HA antibodies. The filter stained with Ponceau S (Pon. S) was used as loading control. Asterisk indicates unspecific bands.

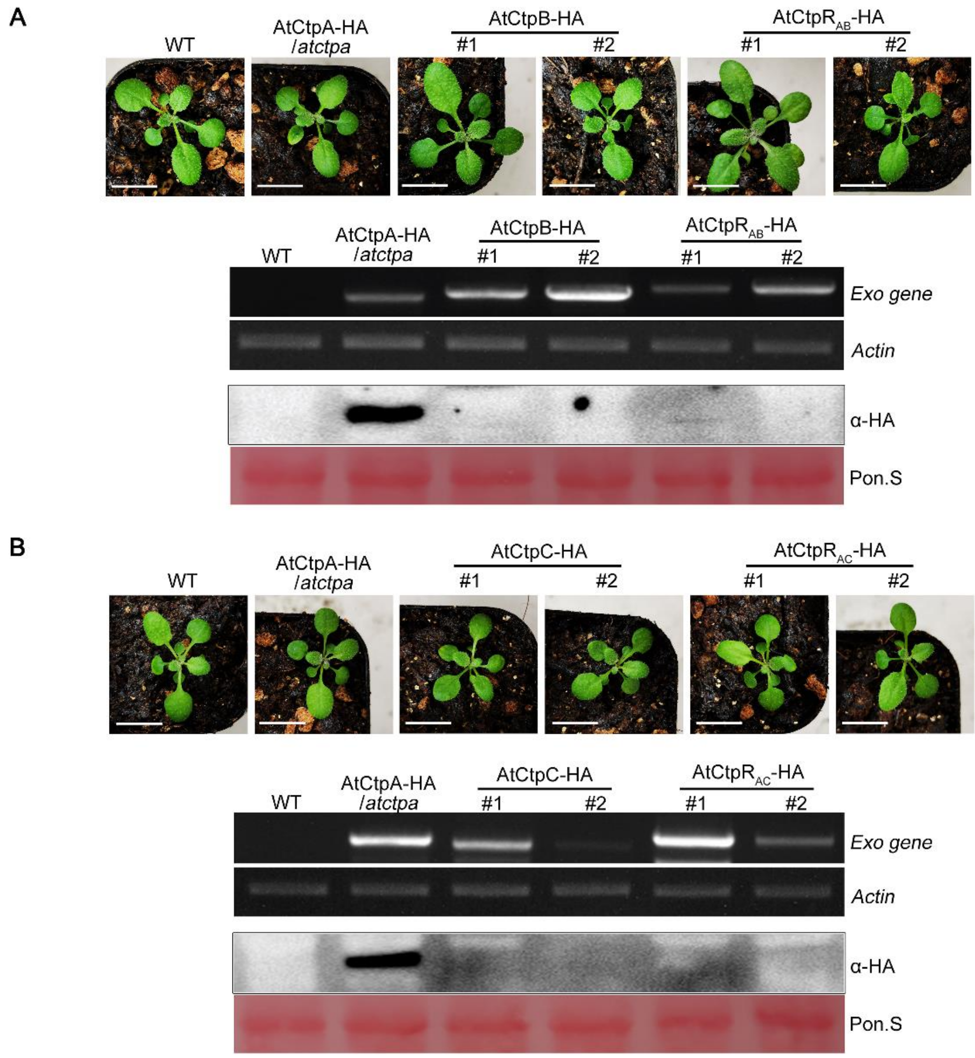

Supplementary Figure S5. Characterizations of transgenic plants carrying *AtCtpB* or *R_{AB}* and *AtCtpC* or *R_{AC}* driven by 35S promoter in wild type background.

AtCtpB-HA or *AtCtpR_{AB}-HA* and *AtCtpC-HA* or *AtCtpR_{AC}-HA,* driven by CAMV 35S, were transformed into wild type (WT) Columbia through agrobacteria mediated method, and the diagrams for them were the same as Figure 6A. The upper panels in (A) and (B): T2 transgenic plants grown in soil under NL light (80 μmol/s/m^2) for two weeks. #1 and #2 represent two independent lines. Scale bars indicate 1 cm. The lower panels in (A) and (B): the expressions of exogenous transgenes (*Exo*) in transgenic

plants were analyzed at RNA (top two images) and protein (bottom two images) levels by semi qRT-PCR and immunoblot. Native *Actin 2* (*AT3G18780*) and the filters stained with Ponceau S (Pon. S) were used as quantity controls, respectively.

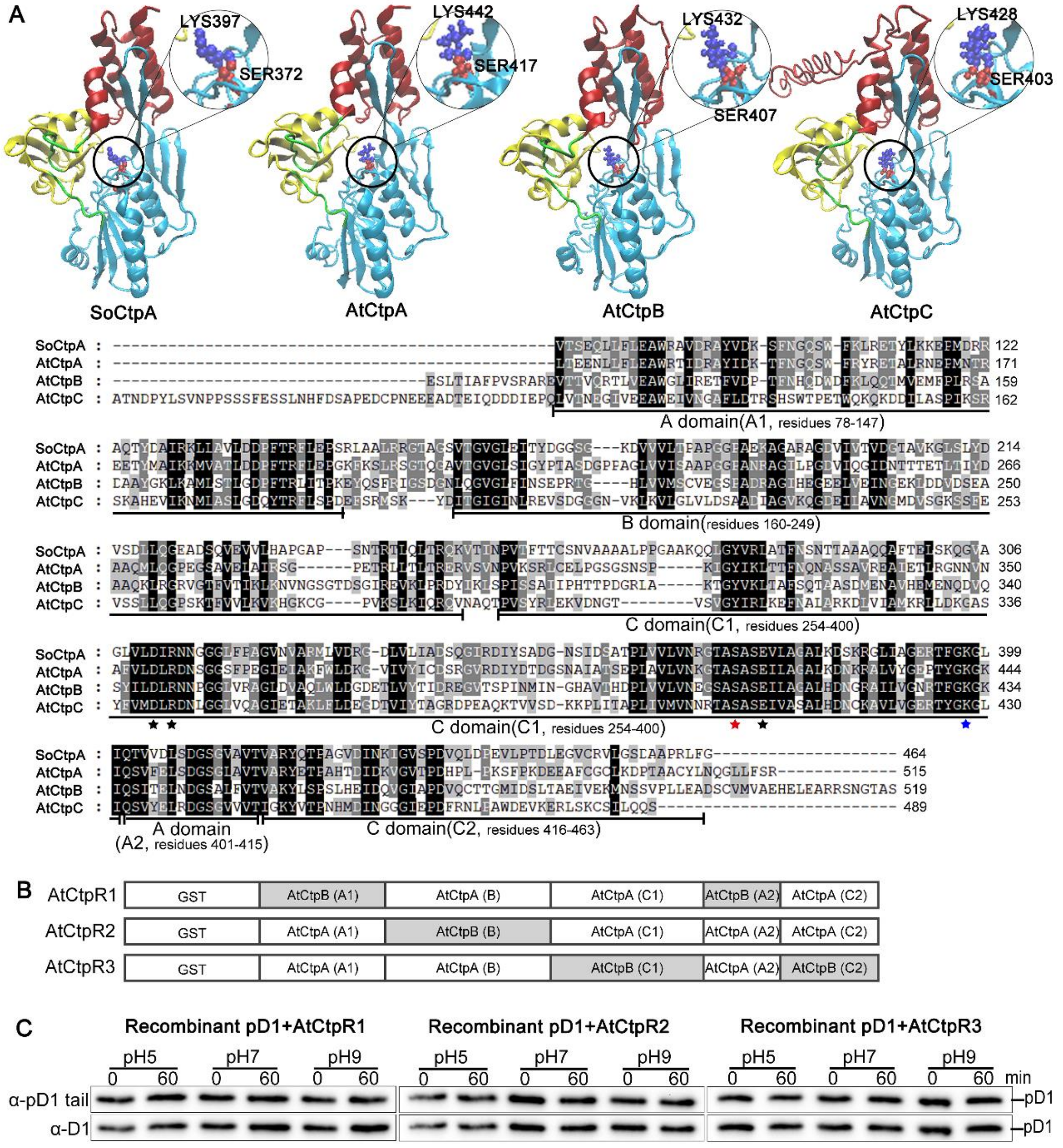

Supplementary Figure S6. Domain swapping Analysis of AtctpA and ActpB.

(A) The structure (upper) and sequence (lower) comparisons of SoCtpA, AtCtpA, AtCtpB, and AtCtpC. The tertiary structure of *Scenedesmus obliquus* CtpA (SoCtpA) was obtained from PDB database (https://www.rcsb.org/structure/1fcf). The 3D structures of AtCtpA, AtCtpB, and AtCtpC were predicted by I-TASSER (Roy et al., 2012; Yang and Zhang, 2015; Zhang et al., 2017; Zhang, 2009). These four pdb files were uploaded in the Visual Molecular Dynamics program (Humphrey et al., 1996) to generate the presented structures. A domain is colored in red, B domain in yellow, C domain in blue, and loops between domains in green. The amino acids of catalytic dyad in structures were colored in blue (Lys) and red (Ser) as showed by magnification. Sequence comparisons were performed with ClustalW program in MEGA6. The structural domains are marked with black straight lines below the

corresponding sequences. The conserved amino acids essential for catalytic activity of CtpA (Inagaki et al., 2001; Che et al. 2013) are indicated by asterisks, in which Lys and Ser composing the catalytic dyad are marked with blue and red asterisks, respectively.

(B) Schematic diagrams for domain swapping experiment between AtCtpA and AtCtpB. The A, B, and C domains in GST fused AtCtpA were respectively replaced by their counterparts in AtCtpB (grey boxes) to form recombinant proteins AtCtpR1, AtCtpR2, and AtCtpR3. Un-swapped domains in each protein were drawn in white boxes.

(C) *In vitro* protease activity tests of AtCtpR1, AtCtpR2, and AtCtpR3 were carried out by using synthesized pD1 substrate as that in figure 4.

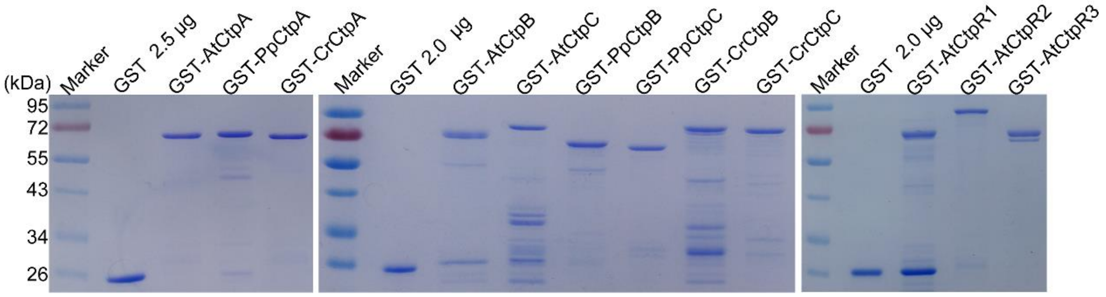

Supplementary Figure S7. SDS-PAGE analyses of Ctp proteins used in the protease activity assays.

After purification, the amounts of GST-Ctps in eluents were evaluated by SDS-PAGE analyses relative to standard GST protein. Ten μg of Ctp proteins were used in subsequent protease activity assays.

Supplementary Table 1 Protein accession numbers in phylogenetic tree analyses.

pD1 Type	Organism	CtpA				CtpB				CtpC				Database
		**Accession number**	coverage (%)	Score	E-value	**Accession number**	coverage (%)	Score	E-value	**Accession number**	coverage (%)	Score	E-value	
**Group I (16)**	**Cyanophyta (Prokaryotic)**													
	Synechocystis sp. PCC 6803	WP_010873417	—	—	—	WP_010871361	—	—	—	WP_010872128	—	—	—	NCBI
	Synechococcus elongatus PCC 7942	ABB58360.1	88	480	3E-170	ABB56548.1	82	365	4E-124	ABB56744.1	100	543	0	NCBI
	Leptolyngbya boryana	WP_017287738.1	86	458	3E-161	WP_017289088.1	84	417	5E-144	WP_026148389.1	98	567	0	NCBI
	Geitlerinema sp. PCC 7407	WP_015173572.1	97	494	2E-176	WP_015172436.1	93	431	1E-150	WP_015170194.1	98	536	0	NCBI
	Leptolyngbya ohadii	WP_088893494.1	96	474	2E-167	WP_088894311.1	82	390	2E-133	WP_088892848.1	97	531	0	NCBI
	Pleurocapsa minor	WP_015144827.1	95	565	0	WP_015143772.1	84	532	0	WP_015142506.1	99	639	0	NCBI
	Limnoraphis robusta CS-951	KKD37180.1	95	499	1E-177	KKD35691.1	85	412	5E-142	KKD37108.1	98	545	0	NCBI
	Rippkaea orientalis	WP_012593970.1	96	564	0	WP_012594914.1	88	563	0	WP_15957337.1	98	634	0	NCBI
	Gloeocapsa sp. PCC 7428	WP_015188917.1	90	493	7E-175	WP_015186501.1	82	419	5E-145	WP_015190966.1	98	557	0	NCBI
	Oscillatoriales cyanobacterium JSC-12	WP_009555551.1	95	471	2E-166	WP_009555425.1	82	425	2E-147	WP_009557210.1	98	559	0	NCBI
	Leptolyngbya sp. PCC 6406	WP_027268870.1	96	471	1E-166	WP_071992215.1	82	367	9E-125	WP_008316952.1	99	530	0	NCBI

	Chroogloeocystis siderophila	WP_073548808.1	90	489	8E-174	WP_073549695.1	82	419	4E-145	WP_073548177.1	98	559	0	NCBI
	Planktothrix tepida	WP_072717855.1	95	503	5E-179	WP_072717382.1	82	420	2E-145	WP_083580024.1	95	551	0	NCBI
	Oscillatoria sp. PCC 10802	WP_017716499.1	95	495	8E-176	WP_017717799.1	82	430	2E-149	WP_026098110.1	97	555	0	NCBI
	Oscillatoriales cyanobacterium CG2_30_40_61	OIP73180.1	95	500	4E-178	OIP67295.1	82	419	2E-145	OIP68024.1	99	561	0	NCBI
	Planktothrix sp. PCC 11201	WP_079679774.1	95	507	1E-180	WP_079678415.1	82	416	9E-144	WP_079680804.1	95	547	0	NCBI
	Planktothrix agardhii	WP_042153271.1	95	490	4E-173	WP_042155587.1	82	417	7E-144	WP_042154560.1	99	558	0	NCBI
	Planktothrix paucivesiculata PCC 9631	VXD20545.1	95	502	1E-178	VXD25008.1	82	425	2E-147	VXD16497.1	99	560	0	NCBI
	Roseofilum reptotaenium AO1-A	OJJ24040.1	93	472	6E-167	OJJ25102.1	94	406	3E-140	OJJ25011.1	95	524	0	NCBI
	Phormidium ambiguum	WP_073593457.1	90	490	5E-174	WP_073591607.1	82	364	2E-123	—	—	—	—	NCBI
**Group II (9)**	**Chlorophyta (Eukaryotic)**													
	Chlamydomonas reinhardtii	Cre10.g420550.t1.1	96	357	1.4E-118	Cre10.g453807.t2.1	96	355	4E-116	Cre06.g265850.t1.1	93	267	1.2E-80	JGI
						Cre10.g426050.t1.2	79	229	3E-40	Cre17.g739150.t1.2	83	261	5E-30	JGI
	Dunaliella salina	Dusal.0111s00010.1	96	359	1.1E-119	Dusal.0015s00042.1	83	241	1.4E-74	Dusal.1339s00002.1	88	289	4E-91	JGI
	Volvox carteri		96	372	5.3E-124	Vocar.0030s0105.1	96	355	3E-116	Vocar.0002s0139.1	93	241	9E-71	JGI

	Vocar.0016s0059.2												
					Vocar.0005s0358.1	76	278	4E-41	Vocar.0003s0289.1	74	183	3E-32	JGI
Coccomyxa subellipsoidea C-169	XP_005648207.1	94	413	1.3E-141	XP_005645927.1	98	372.9	2E-125	XP_005652247.1	98	292	3.6E-94	NCBI
					XP_005648181.1	94	197	4.9E-58	XP_005647465.1	85	125	1.3E-32	NCBI
Micromonas pusilla	XP_003056496.1	94	364	1.1E-121	—	—	—	—	XP_003063156.1	87	271	4.5E-87	NCBI
Micromonas sp. RCC299	XP_002502055.1	95	364	1.2E-121	—	—	—	—	XP_002506449.1	94	310	9.1E-101	NCBI
Ostreococcus lucimarinus	XP_001415400.1	96	352	1.5E-118	—	—	—	—	XP_001417628.1	89	305	5.1E-99	NCBI
Scenedesmus obliquus	O04073.1	95	392	1E-136	—	—	—	—	—	—	—	—	NCBI
Monoraphidium neglectum	XP_013900651.1	97	388	1E-133	XP_013901188.1	85	298	1E-97	—	—	—	—	NCBI
Micractinium conductrix	PSC76991.1	94	386	6E-132	PSC70752.1	93	315	2E-100	PSC70735.1	88	328	1E-108	NCBI
Chlorella sorokiniana	PRW34015.1	91	382	1E-126	PRW57074.1	99	323	3E-106	PRW57137.1	86	320	3E-98	NCBI
Chlorella variabilis	XP_005849285.1	94	363	1E-124	XP_005847884.1	97	341	2E-113	XP_005847877.1	83	282	5E-91	NCBI
Chlamydomonas eustigma	GAX73199.1	96	365	9E-124	GAX76907.1	88	332	3E-109	GAX80856.1	90	290	1E-92	NCBI
Raphidocelis subcapitata	GBF95448.1	85	356	3E-120	GBF96323.1	88	306	1E-99	GBF93599.1	86	267	9E-83	NCBI
									GBF93173.1	83	184	5E-51	NCBI
Ostreococcus tauri	XP_003074135.1	95	347	5E-117	—	—	—	—	XP_022838876.1	93	314	5E-104	NCBI
Bathycoccus prasinos	XP_007515489.1	92	326	1E-109	—	—	—	—	XP_007511360.1	90	321	1E-104	NCBI
Auxenochlorella	XP_011396732.1	94	308	1E-102	XP_011397739.1	96	286	2E-86	XP_011399412.1	86	237	2E-70	NCBI

protothecoides													
Gonium pectorale	—	—	—	—	KXZ54634.1	88	337	1E-111	KXZ42218.1	86	273	5E-86	NCBI
**Embryophyte (Eukaryotic）**													
**Bryophyta**													
Physcomitrella patens	XP_024385318.1	100	548	0	XP_024391049.1	97	520	0	XP_024398460.1	97	437	1.2E-148	NCBI
									XP_024376785.1	93	440	2.5E-149	NCBI
									XP_024384517.1	82	212	1.7E-54	NCBI
Marchantia polymorpha	PTQ45047.1	94	542	0	OAE24478.1	97	510	9E-177	PTQ38413.1	95	453	7E-154	NCBI
									PTQ29718.1	84	211	2.1E-47	NCBI
Sphagnum fallax	Sphfalx0044s0010.1	100	535	0	Sphfalx0008s0198.1	97	539	0	Sphfalx0014s0014.2	94	457	4.4E-158	JGI
					Sphfalx0162s0014.1	97	513	2E-178	Sphfalx0020s0021.1	83	166	3.3E-45	JGI
**Tracheophyte**													
Selaginella moellendorffii	XP_002979781.1	100	509	6.6E-180	XP_024540786.1	96	485	7E-170	XP_002965587.1	86	448	4E-160	NCBI
**Angiosperm**													
Amborella trichopoda	XP_011625922.1	100	619	0	XP_006850171.1	100	659	0	XP_011621275.1	83	429	2.2E-147	NCBI
**Monocots**													
Ananas comosus	XP_020112923.1	100	608	0	XP_020090596.1	100	498	1E-171	XP_020098589.1	86	393	4.5E-133	NCBI
Musa acuminata	XP_009407843.1	100	630	0	XP_009383212.1	100	630	0	XP_009406283.1	97	542	0	NCBI
Spirodela polyrhiza	Spipo1G0011200	100	635	0	Spipo12G0033700	100	637	0	Spipo14G0041900	94	529	0	JGI
Zostera marina	KMZ68881.1	100	602	0	KMZ60132.1	98	577	0	KMZ70100.1	92	516	0	NCBI
Brachypodium	XP_003578617.1	100	632	0	XP_003563904.1	100	618	0	XP_003569507.1	90	513	0	NCBI

distachyon3													
Brachypodium stacei3	Brast05G210100.1	100	632	0	Brast07G109600.1	100	617	0	Brast01G192500.1	90	515	1.4E-180	JGI
Oryza sativa Japonica Group	XP_015626598.1	100	622	0	XP_015643584.1	100	620	0	XP_015622457.1	92	552	0	NCBI
Oropetium thomaeum	Oropetium_20150105_04627A	75	446	2.7E-156	Oropetium_20150105_23514A	100	618	0	Oropetium_20150105_11691A	82	369	1.6E-124	JGI
Panium hallii	Pahal.A03864.2	100	634	0	Pahal.D02099.1	100	625	0	Pahal.E02231.1	92	529	0	JGI
Panicum virgatum	Pavir.Ab03278.1	100	637	0	Pavir.Da01617.1	100	623	0	Pavir.Eb02683.1	90	521	0	JGI
	Pavir.Aa00118.1	91	637	0					Pavir.Ea02341.1	71	353	7.9E-120	JGI
Setaria italica	Seita.1G364200.1	100	630	0	Seita.4G138700.1	100	621	0	—	—	—	—	JGI
Sorghum bicolor	Sobic.004G343500.1	100	626	0	Sobic.010G131700.1	100	618	0	Sobic.003G247400.2	92	515	0	JGI
Zea mays PH207	Zm00008a023329_T01	100	630	0	Zm00008a034149_T01	100	591	0	Zm00008a032213_T01	90	519	0	JGI
**Eudicot**													
Aquilegia coerulea	Aqcoe5G010500.1	100	662	0	Aqcoe1G023500.1	100	671	0	Aqcoe1G203500.1	94	538	0	JGI
Amaranthus hypochondriacus	AHYPO_003918-RA	100	582	0	AHYPO_000094-RA	100	589	0	AHYPO_004375-RA	94	519	0	JGI
Daucus carota	DCAR_002290	100	642	0	DCAR_013578	100	673	0	DCAR_031456	95	480	5E-163	JGI
Mimulus guttatus	—	—	—	—	Migut.J01789.1	100	659	0	Migut.M00953.1	70	351	2.4E-117	JGI
Solanum lycopersicum	Solyc12g097030.1.1	100	651	0	Solyc03g059260.2.1	100	663	0	Solyc02g071190.2.1	87	512	2.8E-179	JGI
Solanumerosum	PGSC0003DMT400022218	100	651	0	PGSC0003DMT400084308	100	670	0	PGSC0003DMT400007927	91	534	0	JGI
Kalanchoe	Kaladp0024s0051.1	100	675	0	Kaladp0087s0188.1	100	672	0	—	—	—	—	JGI

fedtschenkoi													
Kalanchoe laxiflora	Kalax.0130s0052.1	100	673	0	Kalax.0322s0004.1	100	665	0	Kalax.0835s0023.1	93	536	0	JGI
	Kalax.1447s0004.1	84	554	0	Kalax.0637s0013.1	99	660	0	Kalax.0164s0012.3	93	535	0	JGI
Eucalyptus grandis	Eucgr.H02885.1	89	604	0	Eucgr.A02402.1	96	651	0	Eucgr.E02360.3	98	471	1.2E-163	JGI
Vitis vinifera Genoscope	GSVIVT01001855001	100	677	0	GSVIVT01033332001	100	691	0	GSVIVT01021388001	90	563	0	JGI
Linum usitatissimum	Lus10004402	99	600	0	Lus10040494	100	676	0	Lus10004601	88	571	0	JGI
	Lus10023704	99	594	0					Lus10004545	95	439	2.1E-150	JGI
Manihot esculenta	Manes.03G156100.1	100	688	0	Manes.09G029500.1	100	691	0	Manes.17G065400.1	92	575	0	JGI
Populus trichocarpa	Potri.008G173900.4	100	679	0	Potri.006G055400.1	100	674	0	Potri.011G078700.1	95	572	0	JGI
Ricinus commnis	29904.m003051	100	671	0	27810.m000652	85	445	2E-152	29693.m002070	88	556	0	JGI
Salix purpurea	SapurV1A.0021s0290.1	100	683	0	SapurV1A.0133s0220.1	100	664	0	SapurV1A.0031s0280.1	95	560	0	JGI
Citrus sinensis	orange1.1g009668m	100	677	0	orange1.1g047092m	100	662	0	orange1.1g012084m	89	579	0	JGI
Citrus clementina	Ciclev10004718m	100	677	0	Ciclev10004757m	100	679	0	Ciclev10018134m	89	557	0	JGI
Carica papaya	evm.model.supercontig_59.36	100	681	0	evm.model.supercontig_85.132	100	693	0	—	—	—	—	JGI
Gossypium raimondii	Gorai.006G253900.1	100	664	0	Gorai.002G229800.1	100	687	0	Gorai.009G157600.3	89	547	0	JGI
	Gorai.001G131500.1	75	299	6.4E-99									JGI
Theobroma cacao	Thecc1EG021046t1	100	667	0	Thecc1EG024797t1	100	680	0	Thecc1EG029558t1	98	572	0	JGI
Arabidopsis thaliana	AT4G17740.1	—	—	—	AT3G57680.1	—	—	—	AT5G46390.1	—	—	—	JGI

	Boechera stricta	Bostr.30275s0238.1	100	783	0	Bostr.20903s0020.1	100	820	0	Bostr.8819s0177.1	100	759	0	JGI
	Brassica oleracea capitate	Bol037109	100	759	0	Bol045512	100	763	0	Bol032116	95	676	0	JGI
	Brassica rapa FPsc	Brara.K00491.1	100	757	0	Brara.I03985.1	100	786	0	Brara.I01932.1	100	714	0	JGI
	Capsella grandiflora	Cagra.3356s0066.1	100	779	0	Cagra.0210s0005.1	100	817	0	Cagra.0569s0015.1	95	737	0	JGI
	Capsella rubella	Carubv10007107m	100	778	0	Carubv10018381m	100	808	0	Carubv10026297m	95	735	0	JGI
	Eutrema salsugineum	Thhalv10024882m	100	764	0	Thhalv10005897m	98	802	0	Thhalv10001155m	100	727	0	JGI
	Cucumis sativus	Cucsa.383050.2	100	647	0	Cucsa.357250.2	100	683	0	Cucsa.212480.1	95	537	0	JGI
	Fragaria vesca	mrna28308.1-v1.0-hybrid	97	659	0	mrna14365.1-v1.0-hybrid	100	687	0	mrna24893.1-v1.0-hybrid	93	553	0	JGI
	Glycine max Wm82	Glyma.20G199000.1	100	652	0	Glyma.10G094900.1	100	681	0	Glyma.05G165100.5	94	557	0	JGI
	Malus domestica	MDP0000219700	99	661	0	MDP0000305189	100	649	0	—	—	—	—	JGI
		MDP0000251269	100	654	0									JGI
	Medicago truncatula	Medtr1g073130.1	100	653	0	Medtr1g052135.1	100	676	0	Medtr8g088150.1	96	555	0	JGI
	Phaseolus vulgaris	Phvul.L001972.1	99	644	0	Phvul.007G255600.1	100	684	0	Phvul.002G245300.1	93	555	0	JGI
	Prunus persica	Prupe.1G052500.2	100	664	0	Prupe.7G068000.1	100	702	0	Prupe.4G074500.1	94	561	0	JGI
	Trifolium pratense	Tp57577_TGAC_v2_mRNA19200	100	663	0	Tp57577_TGAC_v2_mRNA23564	100	664	0	Tp57577_TGAC_v2_mRNA6999	94	503	4.7E-176	JGI
**Group III (0)**	**Chlorarachniophyta （Eukaryotic）**													
	Bigelowiella natans	—	—	—	—	—	—	—	—	Bigna1|87480	C-87	173	1E-45	JGI
										Bigna1|133319	A-94	125	6E-37	JGI
	**Dinophyta （Eukaryotic）**													

	Symbiodinium microadriaticum	—	—	—	—	—	—	—	—	OLP86183.1	A-89	236	3E-52	NCBI
**Outgroup**	*Escherichia coli*	WP_096246988					MRF42352.1							NCBI

Supplementary Table S2. Primers used in this study.

Names	Primer sequences (5'-3')	Purposes
A-F1	TTCCTTGATGGTGGTTCAGTC	*atctpa* genotyping
A-R1	GCCTCGAGGAAGAGAAGATTC	*atctpa* genotyping
A-F2	AGAGTTGGAATTTTCCCGGGGAA	*AtCtpA* full length cDNA amplification
A-R2	TCATCTAGAAAAAAGTAGGCCTTGATTG	*AtCtpA* full length cDNA amplification
B-F1	ATGGAAAAAGTTGTAACCCTAAATCATG	*AtCtpB* CDS amplification and *atctpb-1* genotyping
B-F2	TGATGGACGTTTAGCGAAGAC	*atctpb-2/-3* genotyping
B-R	TCAAGAAGCTGTTCCATTAGATCGTC	*AtCtpB* CDS amplification and *atctpb* genotyping
C-F	ATGAGGCTTTTGCTTCCATTTTCCTCGC	*AtCtpC* CDS amplification and *atctpc* genotyping
C-R1	GAAACTGGGGTCTGTGCATTA	*atctpc* genotyping
C-R2	TCATCAACTTTGTTGGAGAATACTGCAC	*AtCtpC* CDS amplification
BP1	ATTTTGCCGATTTCGGAAC	SALK line genotyping
BP2	GCCTTTTCAGAAATGGATAAATAGCCTTGCTTCC	SAIL line genotyping
BP3	ATAATAACGCTGCGGACATCTACATTTT	GABI line genotyping
pRI101-AN-F	GATCAAAAGCAAGTTCTTCACTG	Transgene amplification
pRI101-AN-R	AAATTCGAGCTCGGTAGCAATTC	Transgene amplification
TP_{AtCtpA}-R	ACCCCATGATGGTGGTGAATCAG	Transgene amplification
Actin-F	CTCTCCCGCTATGTATGTCGCCATCC	*Actin* gene amplification
Actin-R	CTGTGAACGATTCCTGGACCTGCCTC	*Actin* gene amplification
GAPDH-F	CCATGGCTTCGGTTACTTTC	qRT-PCR of internal control gene
GAPDH-R	CACCTTAAGCTTGGCCTCAG	qRT-PCR of internal control gene
qPCR (CtpA)-F	TTCTGTTCTGCTTGTCTCTTCC	qRT-PCR of *AtCtpA*
qPCR (CtpA)-R	CTCTGTAACGAAACCAGCTTTG	qRT-PCR of *AtCtpA*
qPCR (CtpB)-F	TCGTGAGGTTACTACAGTACAG	qRT-PCR of *AtCtpB*
qPCR (CtpB)-R	AAGCAGCATCTGCTGATCTTAG	qRT-PCR of *AtCtpB*
qPCR (CtpC)-F	GTAGTTCTCAAGGTGAAGCATG	qRT-PCR of *AtCtpC*
qPCR (CtpC)-R	ATCTTTTCTAGCCAGCGCATTG	qRT-PCR of *AtCtpC*
